# BMP-FGF Signaling Axis Mediates Wnt-Induced Epidermal Stratification in Developing Mammalian Skin

**DOI:** 10.1371/journal.pgen.1004687

**Published:** 2014-10-16

**Authors:** Xiao-Jing Zhu, YuDong Liu, Zhong-Min Dai, Xiaoyun Zhang, XueQin Yang, Yan Li, Mengsheng Qiu, Jiang Fu, Wei Hsu, YiPing Chen, Zunyi Zhang

**Affiliations:** 1 Institute of Developmental and Regenerative Biology, College of Life and Environmental Science, Hangzhou Normal University, Zhejiang, China; 2 Key Laboratory of Mammalian Organogenesis and Regeneration, Zhejiang, China; 3 Department of Biomedical Genetics, Center for Oral Biology, James P. Wilmot Cancer Center, University of Rochester Medical Center, Rochester, New York, United States of America; 4 Department of Cell and Molecular Biology, Tulane University, New Orleans, Louisiana, United States of America; University of Lausanne, Switzerland

## Abstract

Epidermal stratification of the mammalian skin requires proliferative basal progenitors to generate intermediate cells that separate from the basal layer and are replaced by post-mitotic cells. Although Wnt signaling has been implicated in this developmental process, the mechanism underlying Wnt-mediated regulation of basal progenitors remains elusive. Here we show that Wnt secreted from proliferative basal cells is not required for their differentiation. However, epidermal production of Wnts is essential for the formation of the spinous layer through modulation of a BMP-FGF signaling cascade in the dermis. The spinous layer defects caused by disruption of Wnt secretion can be restored by transgenically expressed *Bmp4*. Non-cell autonomous BMP4 promotes activation of FGF7 and FGF10 signaling, leading to an increase in proliferative basal cell population. Our findings identify an essential BMP-FGF signaling axis in the dermis that responds to the epidermal Wnts and feedbacks to regulate basal progenitors during epidermal stratification.

## Author Summary

Epidermis, a thin layer of stratified epithelium forming the outmost surface of the skin, provides the crucial function to protect animals from environmental insults, such as bacterial pathogens and water loss. This barrier function is established in embryogenesis, during which single layered epithelial cells differentiate into distinct layers of keratinocytes. Many human genetic diseases are featured with epidermal disruption, affecting at least one in five patients. Skin regeneration and future therapeutics require a thorough understanding of the molecular mechanisms underlying epidermal stratification. Wnt ligands have been implicated in hair follicle induction during skin development and self-renewal of stem cells in the interfollicular epidermis of adult skin; however, little is known about the mechanism of how Wnt signaling controls epidermal stratification during embryogenesis. In this study, by using a genetic mouse model to disrupt Wnt production in skin development, we found that signaling of epidermal Wnt in the dermis initiate mesenchymal responses by activating a Bone Morphogenetic Protein (BMP) and Fibroblast growth factor (FGF) signaling cascade, and this activation is required for feedback regulations in the embryonic epidermis to control stratification. Our findings identify a genetic hierarchy of signaling essential for epidermal-mesenchymal interactions, and promote our understanding of mammalian skin development.

## Introduction

Vertebrate epidermis, the outermost layer of skin, functions as a barrier for protection against environmental insult and dehydration. At approximately embryonic day 8.5 (E8.5) during mouse embryogenesis, the single-layered surface ectoderm adopts an epidermal developmental fate by turning off the expression of keratins 8 and 18 (K8/K18) and switching on the expression of K5/K14, leading to the replacement of the unspecified ectoderm by the embryonic basal layer [1], [2]. Subsequently, the change of cell proliferation from symmetric to asymmetric division becomes evident at E12.5 to 14.5 [3]. The proliferative basal layer periodically produces intermediate suprabasal cells positive for K1/K10, programmed for terminal differentiation of keratinocytes [2]. The transient intermediate keratinocytes then exit the cell cycle, followed by detachment from the basal layer and migration outward to form the spinous layer, characterized by the expression of K1 and K10. Subsequent developmental events engage the expression of differentiation genes, including loricrin and filaggrin, as spinous keratinocytes further develop into the granular and cornified layers contributing to barrier establishment at late embryonic stages ([2].

The tumor-suppressor p53-related transcription factor, p63, encodes regulators required for initiating epithelial stratification during development and maintaining proliferative potential of the basal layer keratinocytes [4], [5], [6], [7]. Two different classes of protein are encoded by p63: the first contains the amino terminal transactivation domain (TA isoforms) and the second lacks this domain (ΔN isoforms) [8]. ΔNp63 is expressed predominantly in the basal layer keratinocytes but its expression is down-regulated in the post-mitotic suprabasal keratinocytes, suggesting that p63 plays a crucial role in proliferative capacity of the epidermal progenitors [9], [10].

Several families of secreted signaling molecules, including bone morphogenetic protein (BMP), fibroblast growth factor (FGF), Hedgehog (Hh), and Wnt, have been implicated in embryonic epidermal morphogenesis. Among them, Wnt appears to be the earliest signal known to promote epidermal development [11], [12], [13]. Our previous studies have demonstrated that embryonic epidermis is the source of Wnts essential for establishing and orchestrating signaling communication between the epidermis and the dermis in hair follicle initiation [14]. Overexpression of Dkk1, a Wnt antagonist, in the epidermis also results in the absence of hair follicles [11], whereas expression of a constitutively active form of β-catenin in the epithelium leads to premature development of the hair follicle placode [15]. In chicks, high levels of Wnt are able to activate BMP signaling through repression of FGF signaling, leading to a switch of neural cell fate into epidermal cell fate [16], [17]. In addition, BMP signals have also been suggested to control p63 expression during ectodermal development [18]. In an embryonic stem cell (ESC) model recapitulating the stepwise appearance of the epidermal stratification in vitro, BMP4 treatment activates the expression of ΔNp63 isoforms, promoting an induction of the proliferative basal keratinocyte makers, K5 and K14, and a progressive enhancement of the terminal differentiation markers, K1, K10, involucrin and filaggrins [19]. In addition, BMP signals have also been suggested to control p63 expression during ectodermal development. Moreover, BMP signaling is also active in the interfollicular epidermis where it may act as a morphogen by promoting epidermal development through inhibition of the hair follicle fate during skin morphogenesis [1], [11], [20], [21]. It has been suggested that FGF7 (KGF) and FGF10 function in concert via FGFR-2 (IIIb) to stimulate keratinocyte proliferation in the epidermis [22], [23], [24], [25], [26], despite the fact that targeted loss of *Fgf7* has no effect on skin development in the mouse [27]. Interestingly, FGF ligands appear to be expressed in the dermis while the receptor is present in the epidermis during skin development [22], [24], [28]. However, how these developmental signals are integrated and interplayed across the epithelium and mesenchyme to control epidermal stratification remains to be elucidated.

In this study, we investigated the genetic regulation of these signaling pathways during epidermal stratification and elucidated the mechanism underlying this developmental process orchestrated by the Wnt, BMP, and FGF signaling pathways. Using a mouse model with epithelial ablation of *Gpr177* (also known as *Wls/Evi/Srt* in *Drosophila*), a regulator essential for intracellular Wnt trafficking, to disrupt Wnt secretion in skin development [29], [30], [31], [32], we identified a crucial role of Wnt signaling in orchestrating epidermal stratification. We demonstrate that signaling of epidermal Wnt to the dermis initiates mesenchymal responses by activating a BMP-FGF signaling cascade. This activation is required for feedback regulations in the epidermis to control the stratification process. Our findings thus decipher a hierarchy of signaling loop essential for epithelial-mesenchymal interactions in the mammalian skin development.

## Results

### Epithelial Wnt secretion mediated by Gpr177 is essential for epidermal development

**Figure 1:**
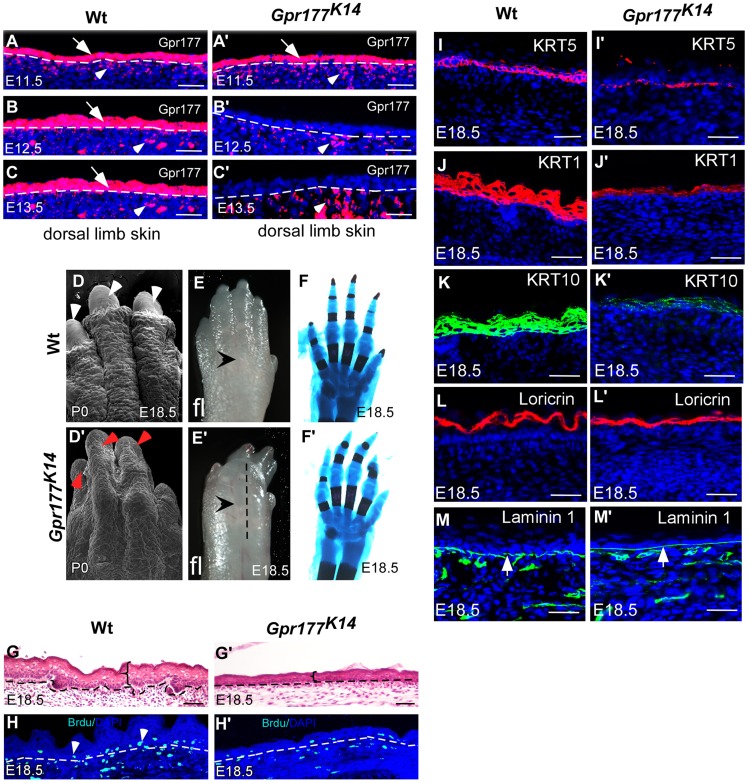
Deletion of *Gpr177* in embryonic epidermis results in skin defects attributing to hypoplastic spinous layer. (A–C, A′–C′) Immunofluorscence (red) of *Gpr177* expression in epidermis (arrows) and underling dermis (arrowheads) in dorsal skin of embryonic limb between E11.5 and E13.5. Note that in *Gpr177^K14^* mutant, *K14-Cre* deleted Gpr177 specifically in epidermis of limb at E12.5 and E13.5 (B′ and C′). (D–E, D′–E′) Scanning electronic microscopic images show loss of nail and lack of skin wrinkles in the *Gpr177^K14^* limb. Note obvious edematous limb surface in the mutants (E′) as compared to wild type controls (E). (F, F′) Skeletal staining by Alcian blue (blue) and Alizarian Red (red) shows comparable skeletal patterning in the *Gpr177^K14^* and control limbs (C and F). (G, G′) H&E staining shows the hypoplastic limb skin of *Gpr177^K14^* mice at E18.5. (H, H′) BrdU incorporation assay shows the cell proliferation in the *Gpr177^K14^* limb skin. (I–M, I′–M′) Immunohistochemistry shows expression of KRT5 (red) for basal cells, KRT1 (red) and KRT10 (green) for spinous layer, Loricrin for granular layer (red), and laminin 1 (green) for the presence of basal membrane (green). Bars: 50 µm.

Gpr177 is expressed in the skin of the developing limb bud as early as E11.5 (Figure S1A, B). Similar to our previous observations in dorsal body skin [14], Gpr177 protein can be found predominantly in the epidermis and weakly in the underlying dermis (Figure 1A–C) at E11.5–13.5. To assess the requirement of epidermal Wnts in the development of skin, we generated *Gpr177^K14^* mice in which *Gpr177* is inactivated by the *K14-Cre* transgenic allele to disrupt the secretion of Wnt proteins [32]. Using a R26R reporter line, we examined the Cre-mediated deletion, which occurs only in the epidermis (Figure S1C, D). The loss of *Gpr177* was clearly evident in the epidermis but not the dermis of *Gpr177^K14^* (Figure 1A′–C′), indicating a targeted removal of *Gpr177* in the mutants. We noted that the Cre recombination is uniformly detected in the limb skin (Figure S1C, D) but not in the dorsal body skin (Figure S1 E, F–G, F′–G′) using the *K14-Cre* line. Compared to the *Gpr177^K5^* mice that exhibited a uniform expression pattern of Cre and consistent phenotypes associated the *Gpr177* deletion described previously [14], the *Gpr177^K14^* mice are not suitable for the study of the body skin due to inconsistent results on skin thickness (Figure S1 F–H, F′–H′). However, the *Gpr177^K14^* model is ideally suited for studies on epidermal development of the limb.

The *Gpr177^K14^* autopods displayed severe deformities including loss of nail formation (Figure 1D, D′). The interdigital and dorsal soft tissues appeared to be edematous (Figure 1E, E′), but skeletal staining revealed comparable structures between controls and mutants (Figure 1F, F′), suggesting that the dysmorphic features of the *Gpr177^K14^* autopods is likely due to impairments in the skin tissue. Histological analysis of autopods showed a reduction in skin thickness as well as in cell proliferation rate, indicating the ablation of skin stratification in *Gpr177^K14^* (Figure 1G–H′ and Figure S2A, B and E). To further investigate the edematous skin abnormalities, we characterized epidermal stratification of the limb skin using markers specific for basal, spinous, and granular epidermal layers. The deletion of *Gpr177* diminished the number of basal cells expressing KRT5 (Figure 1I, I′). Significant reduction of the spinous layer positive for KRT1 and KRT10 was also identified in the longitudinal sections along the dorsal skin of the mutant autopods (Figure 1J–K, J′–K′). However, the granular layer positive for loricrin and the basal membrane protein, laminin 1, did not show significant alterations (Figure 1L–M, L′–M′). The results were consistent with alterations of the limb skin thickness caused by the Cre-mediated deletion of *Gpr177* (Figure S2). Besides, an uneven decrease in skin thickness also occurred in the dorsal body of *Gpr177^K14^*, as shown by histology (Figure S1H, H′) and immunohistochemistry specific for the spinous and basal layers (Figure S1I–J, I′–J′). TUNEL assay did not reveal significant changes in apoptosis, indicating that defects in the spinous layer were not caused by abnormal cell death (Figure S3). Thus, the spinous hypoplasia is likely attributed to defects in the epithelial vertical expansion of *Gpr177^K14^* mice.

### Epidermal deletion of *Gpr177* interferes with canonical Wnt signaling in the underlying dermis

**Figure 2:**
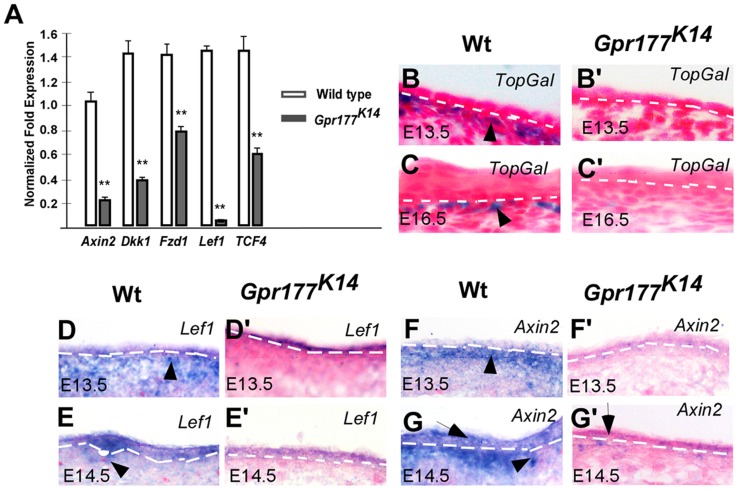
Deletion of Gpr177 in embryonic epidermis leads to ablation of Wnt/β-catenin signaling in dermis. (A) Quantitative RT-PCR shows a decrease in the expression of *Axin2, Dkk1, Fzd1, Lef1, TCF4* in limb skin of mutants at E16.5. **, P<0.01, compared to wild type controls. Data are represented as mean ± SD and are representatives of at least three independent experiments. (B–C, B′–C′) X-Gal staining on sections of dorsal limbs shows lack of TopGal activity in dermal mesenchyme at E13.5 and E16.5 in *Gpr177^K14^*, as compared to wild type controls (Wt). (D–G, D′–G′) In situ hybridization on the sections of embryonic limb skin shows the down-regulation of *Axin2* and *Lef1* in embryonic epidermal stratification of mutants at E13.5 and E14.5.

The deletion of *Gpr177* has been shown to affect Wnt signaling during the development of other organs [14], [32], [33]. This is also true during the morphogenesis of the limb skin, as the the expression of several downstream mediator critical for Wnt signal transduction including *Axin2*, *Dkk1*, *Fzd1*, *Lef1*, and *TCF4* was significantly reduced in the skin of *Gpr177^K14^* autopods (Figure 2A), and the activity of Wnt/β-catenin signaling in the mesenchyme underlying the interfollicular epithelium was almost completely eliminated, evidenced by the lack of *TopGal* reporter activity (Figure 2B–C, B′–C′). In situ hybridization analysis further confirmed that epidermal ablation of *Gpr177* affects the expression of *Lef1* and *Axin2* in both the epithelium and mesenchyme (Figure 2D–G, D′–G′). These observations are consistent with our observations in dorsal body skin (Figure S4A–D, E–F, E′–F′) [14], indicating a requirement of epidermal Wnt for signaling activation in both epidermal and dermal layers. Consistent with this finding, *Dermo1-Cre* mediated deletion of *Gpr177* in the dermis did not alter the skin thickness (Figure S5), suggesting a dispensable role of dermal Wnt in epidermal stratification.

### Non-cell autonomous requirement of BMP signaling in Gpr177-mediated epidermal stratification

**Figure 3:**
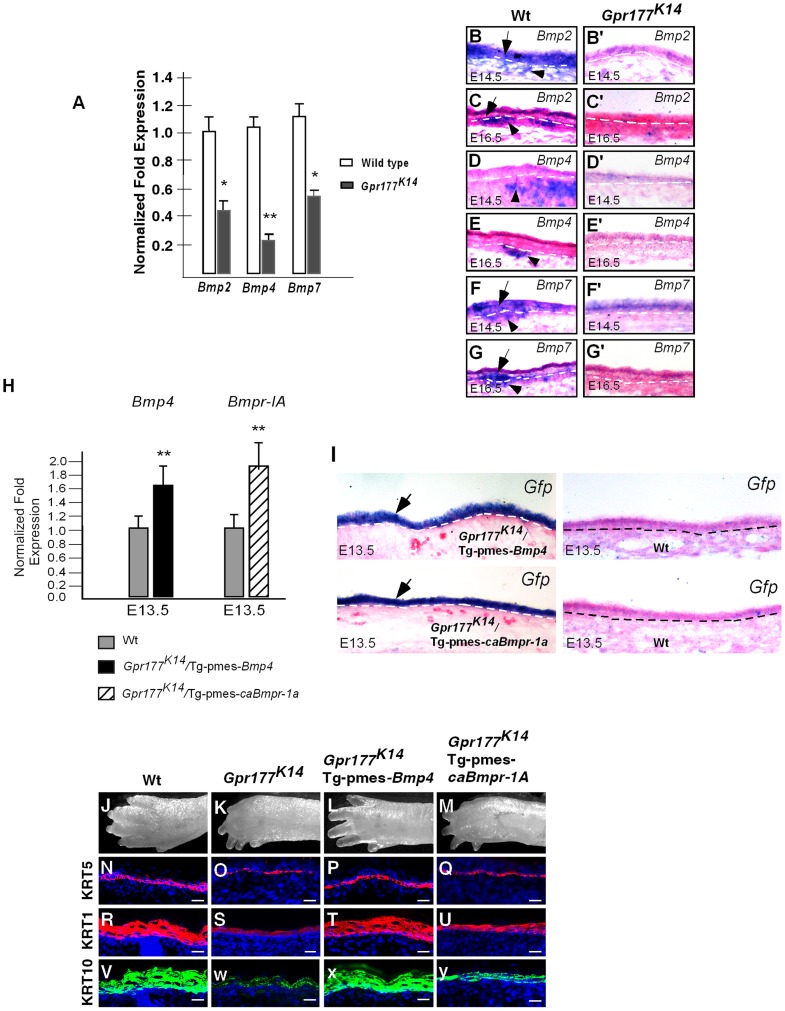
Expression of *Bmps* requires epidermal Gpr177 and rescue of defective limbs and epidermal stratification in *Gpr177^K14^* mutants by activation of a transgenic *Bmp4* allele. (A)Real-time PCR shows the down-regulation of Bmps in skin of *Gpr177^K14^* mutants at E16.5. *,P<0.05; **, P<0.01, compared with Wt. Data are represented as mean ±SD and are representatives of at least three independent experiments. (B–G, B′–G′) In situ hybridization reveals the reduced transcripts of Bmp2, Bmp4, and Bmp7 in epidermis (arrows) and dermis (arrowheads) of *Gpr177^K14^* embryonic limb skin at E14.5 and E16.5, as compared to wild type controls. (H) Quantitative real-time RT-PCR indicates that mRNA levels of *Bmp4* and *Bmp1a* in the autopod skin are enhanced in transgenic animals, compared to wild type controls. Data are represented as mean ± SD (n = 3). *, *P*<0.05, compared with wild type controls. (I) In situ hybridization on *Egfp* mRNA reveals epidermal-specific expression of transgenes in *Gpr177^K14^*/Tg-pmes-*Bmp4* and *Gpr177^K14^*/Tg-pmes-*caBmpr1a*. (J-M) Morphologic defect of autopods in *Gpr177^K14^* mice is partially rescued in *Gpr177^K14^*/Tg-pmes-*Bmp4* mice (J, K, L), but not in *Gpr177^K14^*/Tg-pmes-*Bmpr1a* mice (J, K, M). (N–Y) Immunohistochemistry reveals expressions of KRT5 for basal layer (N–Q), KRT1 and KRT10 for spinous keratinocytes (R–Y). Note that the rescued thickness of spinous layer in *Gpr177^K14^*/Tg-pmes-*Bmp4* (T and X) was comparable to wild type controls (R and V), but not in *Gpr177^K14^*/Tg-pmes-*Bmpr-1a* mice (U and V). Bars: 10 µm.

To decipher the effects of the alteration in Wnt signaling during autopod skin morphogenesis, we performed RNA expression profiling analysis using microarray to identify genes that are differentially expressed in the E15.5 distal limbs (Figure S6 and Table S1 and Table S2). Among those altered genes, members of BMP family were significantly affected in *Gpr177^K14^*. In response to β-catenin/Wnt signaling, BMP signaling in the dermal mesenchyme plays critical role in hair follicle induction [14]. Thus, we hypothesized that BMPs are downstream targets of Wnt signaling and regulate epidermal stratification. Real time RT-PCR analysis validated that *Bmp2*, *4*, and *7* expression was decreased in the mutants (Figure 3A). During normal development of the autopod skin, *Bmp2* and *Bmp7* were found in both the epidermis and dermis while *Bmp4* appeared to be exclusively expressed in the dermis (Figure 3B–G). However, epidermal deletion of *Gpr177* caused profound reduction of *Bmp2*, *Bmp4*, and *Bmp7* in the developing skin (Figure 3B′–G′ and Figure S7A–G, A′–G′), suggesting that BMP signaling, regulated by Wnt signaling, is likely to be involved in epidermal stratification.

To test the functional requirement of BMP signaling in the Gpr177-mediated skin morphogenesis, we used a conditional *Bmp4* transgenic allele. The Tg-pmes-*Bmp4* transgenic mouse was crossed onto the *Gpr177^K14^* background to generate *Gpr177^K14^*/Tg-pmes-*Bmp4* mice. The transgenic *Bmp4* expression from this transgenic allele was tightly controlled by a transcription and translation STOP cassette flanked by two *loxP* sites, permitting the Cre-mediated activation (Figure 3H–I) [34], [35].

The transgenic expression of *Bmp4* was able to alleviate the dysmorphic phenotype caused by the deletion of *Gpr177* (Figure 3J–L). The *Gpr177^K14^*/Tg-pmes-*Bmp4* autopods displayed five separated digits without skin edema (Figure 3L), suggesting that BMP4 acts downstream of Wnt signaling in skin stratification. To determine if this epidermal expression of transgenic *Bmp4* could substitute for mesenchymal *Bmp4* to rescue spinous layer defect, we examined the spinous layer of *Gpr177^K14^*/Tg-pmes-*Bmp4* autopods. Immunostaining of KRT5 and KRT1/10 revealed a significant enhancement in their expression (Figure 3N–P, R–T and V–X). Histological (Figure S2 C–F) and ultrastructural analyses (Figure S2 F–H) further showed that the thickness of the spinous layer was obviously increased in the E18.5 *Gpr177^K14^*/Tg-pmes-*Bmp4* epidermis, as compared to that in *Gpr177^K14^* epidermis.

The transgenic expression of *Bmp4* in the epidermis (Figure 3H–I) may exert its signaling effects in a cell autonomous or non-cell autonomous manner. For non-cell autonomous signaling, it requires the diffusion of BMP4 through an inter-tissue signal transduction mechanism. It has been shown that BMPR1A is responsible for mediating BMP signaling in epidermal development [20], [36], [37]. If the transgenic *Bmp4* indeed acts in a cell autonomous manner, we assumed that activation of BMPR1A-mediated signaling in the epidermis would also alleviate the stratification defects in *Gpr177^K14^* autopods. Accordingly, we compounded a conditional transgenic allele that expresses a constitutively active form of BMPR1A receptor (caBMPR1A) with *Gpr177^K14^* mice (Figure 3H–I) [34]. However, ectopic activation of BMPR1A signaling neither rescued the autopod defects at the morphological (Figure 3M) and histological (Figure S2D) levels nor restored the expression of the basal and spinous layer makers, KRT5 (Figure 3Q), KRT1 (Figure 3U), and KRT10 (Figure 3V), as compared to that in *Gpr177^K14^* mice (Figure 3O, S, W). These results thus suggest a non-cell autonomous BMP signaling across tissue layers to alleviate the epidermal defects of *Gpr177^K14^*, and the BMP4 activity in the dermal mesenchyme, but not in the epidermis, is required for proper stratification of the mammalian skin.

### The role of Wnt/BMP regulatory axis in the development of suprabasal keratinocytes

**Figure 4:**
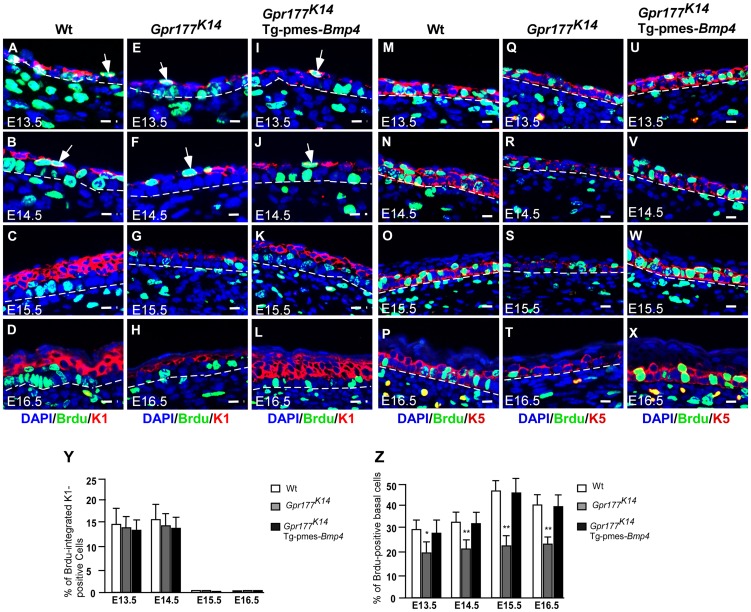
The number of proliferating basal cells is affected in the *Gpr177* mutant, which is rescued by transgenic *Bmp4* expression. (A–L) Immunofluorescence analysis for antibodies against KRT1 (red) and BrdU (green) on sections of dorsal skin of autopods between E13.5 and E16.5. At E13.5 and E14.5, BrdU labeled cells (green) are seen in the first transient suprabasal intermediate cells expressing KRT1 (white arrows in A,B). At E15.5 to E16.5, these cells are replaced by post-mitotic KRT1 cells when spinous layers form as multi-tier keratinocytes where KRT1 positive keratinocytes withdraw from cell cycle (C,D). These processes are comparable among three genotypes (E–H and I–L). Bars: 10 µm. (M–X) Immunofluorscence with antibodies against KRT5 (red) and BrdU (green) on sections of autopod skin. The ratio of BrdU integration in basal layer (M–P for wild type) is reduced in *Gpr177^K14^* (Q–T) during the epidermal stratification. Overexpression of Tg-pmes-*Bmp4* results in an increased ratio of BrdU incorporation in basal layer (U–X). Bars: 10 µm. Dash lines demarcate the boundary between epidermis and dermis. (Y) Statistical analysis shows similar ratios of BrdU incorporation in KRT-1 positive cells in wild type (n = 7), *Gpr177^K14^* (n = 7), and *Gpr177^K14^*/Tg-pmes-*Bmp4* (n = 7) mice during epidermal stratification. Data are represented as mean ± SD. (Z) Percentage of BrdU incorporated KRT-5 cells in embryonic epidermis of wild type (n = 7), *Gpr177^K14^* (n = 7), and *Gpr177^K14^*/Tg-pmes-*Bmp4* (n = 7) mice during epidermal stratification. Data are represented as means ± SD. *, P<0.05; **, P<0.01, compared with wild type controls.

Maturation of the spinous layer first requires the mitotic suprabasal intermediate cells to be replaced by the post-mitotic cells [2]. The hypoplasia developed in the *Gpr177^K14^* spinous layer might be attributed to failure in this replacement. To test this possibility, we performed a BrdU labeling experiment to identify the KRT1 positive keratinocytes undergoing active proliferation between E13.5 and 16.5. Double labeling was able to detect cells positive for BrdU and KRT1 in the E13.5 and 14.5 wild type epidermis (Figure 4A, B). No double positive cells were found at E15.5 and 16.5 (Figure 4C, D). In addition, this replacement process did not seem to be affected by *Gpr177* deletion or transgenic Bmp4 expression (Figure 4E–L and Y). Thus, the initial programming of intermediate cells to become spinous keratinocytes is independent of the Gpr177 mediated regulation and BMP signaling.

As skin stratification requires proper proliferation of the basal cells [9], [10], we further examined if defects in basal cell division contribute to the epidermal abnormalities caused by *Gpr177* deficiency. Double labeling of BrdU and KRT5 permits quantification of the ratio of basal cells proliferation. Closer examinations revealed that the number of KRT5-positive basal cells labeled with BrdU (Figure 4M–P) is significantly reduced by *Gpr177* ablation (Figure 4Q–T). However, this hypoplastic feature was alleviated in the *Gpr177^K14^*/Tg-pmes-*Bmp4* mutants (Figure 4U–X), where the ratio of BrdU labelled basal cells arises between E14.5 and 16.5 to the levels close to controls (Figure 4Z). These observations suggest that the Gpr177-mediated regulation of BMP signaling maintains the high proliferative potential of the basal cells essential for epidermal stratification.

### BMP4 activates basal cell proliferation through modulation of ΔNp63

**Figure 5:**
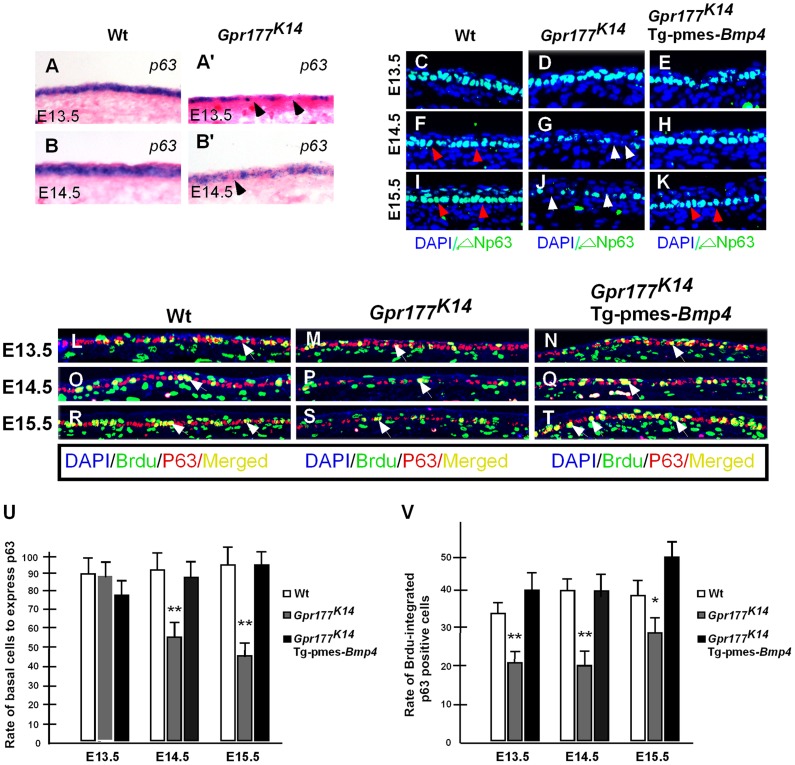
Transgenically expressed *Bmp4* in *Gpr177^K14^* mutants rescued the defective p63 expression. (A–B, A′–B′) In situ hybridization shows *p63* expression in the autopod skin of wild type and *Gpr177^K14^* mice. (C–K) Immunoflurescence with antibody against ΔNp63 in epidermis between E13.5 and E15.5. Note that the reduction of ΔNp63 in *Gpr177^K14^* mutant is restored in *Gpr177^K14^*/Tg-pmes-*Bmp4* mice (arrowheads). (L–T) Immunofluorescence with antibodies against pan-p63 (red) and BrdU (green) reveals BrdU incorporation rate in p63 expressing basal cells. Note that significantly restored merged staining in *Gpr177^K14^*/Tg-pmes-*Bmp4* mice (white arrows) at E14.5 and E15.5. (U) Statistical analysis shows the ratios of p63-positive cells in basal layer cells in wild type control (n = 6), *Gpr177^K14^* mutant (n = 7), and *Gpr177^K14^*/Tg-pmes-*Bmp4* mice (n = 7). Data are represented as mean ± SD. **, P<0.01, compared with wild type controls. (V) Statistical analysis shows percentage of BrdU incorporation in p63 expressing cells in wild type control (n = 7), *Gpr177^K14^* mutant (n = 6), and *Gpr177^K14^*/Tg-pmes-*Bmp4* mice (n = 6). Data are represented as mean ± SD. *P<0.05; **, P<0.01, compared with wild type controls.

It has been shown that p63 transcription factor is critical for the proliferative potential of epidermal stem cells in the stratified epithelium [9], [10], [18], [38]. We therefore tested if p63 is involved in the epidermal stratification mediated by the Wnt/BMP regulatory axis. In situ hybridization analysis showed that the expression of *p63* in the epidermis was affected by *Gpr177* deletion at E13.5 and 14.5 (Figure 5A–B, A′–B′). The loss of *p63* transcripts in the mutants suggests a role of Wnt signaling in the maintenance of its expression in the basal cells (Figure 5A′–B′ and Figure S8A). We next examined the alteration of p63 at the protein level using antibodies against total p63 and its specific isoforms, TA-p63 and ΔNp63. The percentage of the total p63 and ΔNp63 positive basal cells was significantly decreased in *Gpr177^K14^* mutants (Figure 5C–D, F–G, I–J and Figure S8B–J). Consistent with the previous reports [4], [5]. TA-p63 was not involved in epidermal development at these stages (Figure S8 K–P). In addition, transgenic BMP4 was able to elevate the percentage of the total p63 and ΔNp63 positive cells in the basal layer similar to that of wild type control at E13.5, 14.5 and 16.5 (Figure 5C–K and U). To further determine the role of p63 in basal cell proliferation, we performed double labeling of BrdU and p63. The number of the p63-expressing mitotic keratinocytes was reduced in the *Gpr177^K14^* basal layer (Figure 5L–M, O–P and R–S and V), but this reduction was restored by transgenically expressed BMP4 (Figure 5N, Q, T and V), suggesting an involvement of p63 in maintaining the high proliferative potential of basal cells mediated by the Wnt/BMP regulatory axis during epidermal stratification.

### BMP signaling induces epidermal stratification through activation of Smad1/5/8 pathway in the dermis

**Figure 6:**
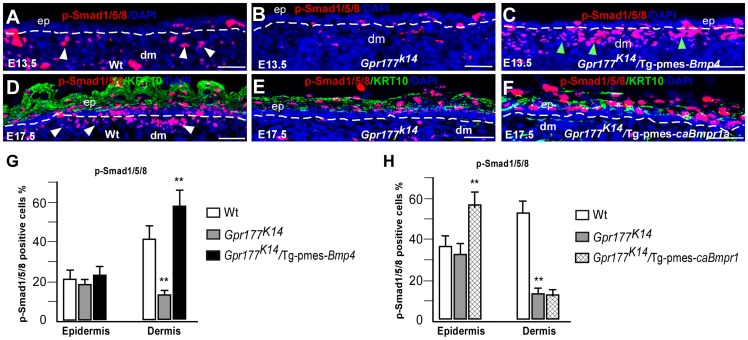
Transgenic pmes-*Bmp4* reactivates Smad1/5/8 signaling in the dermal mesenchyme in *Gpr177^K14^*/Tg-pmes-*Bmp4*. (A–F) Immunofluorescence detections for anti-phosphorylated-Smad1/5/8 (p-Smad1/5/8, red) on sections of autopods. P-Smad1/5/8 activity (white arrowheads) is preferentially decreased in dermis of limb skin in *Gpr177^K14^* mice and increased in dermis of *Gpr177^K14^*/Tg-pmes-*Bmp4* mice (green arrowheads) (A–C). Dash lines demarcate the border of epidermis and dermal mesenchyme. Immunofluorescence staining using antibodies against p-Smad1/5/8 (red) and KRT10 (green) on sections of dorsal autopod skin shows that p-Smad1/5/8 activity is only increased in epidermis of *Gpr177^K14^*/Tg-pmes-*caBmpr-1a* mice (D–F). b: basal layer; ep: epidermis; dm: dermis. Bars: 50 µm. (G–H). Quantification of pSmad1/5/8 positive cells in the epidermis and dermis of *Gpr177^K14^*/Tg-pmes-*Bmp4* (G) and *Gpr177^K14^*/Tg-pmes-*caBmpr-1a* mice (H). Data are represented as mean ± SD. (**, *P*<0.01, n = 3–5).

To further elucidate the mechanism underlying epidermal stratification mediated by BMP signaling, we examined the activation of Smad1/5/8 mediators that transduce the BMP canonical pathway. Immunostaining of phosphorylated Smad1/5/8 revealed that their activations were significantly affected in the dermis, but not in the epidermis of *Gpr177^K14^* mice (Figure 6A, B, G and D, E, H). The dermal-specific effect was restored by transgenically expressed *Bmp4* in *Gpr177^K14^*/Tg-pmes-*Bmp4* mutants (Figure 3H–I, Figure 6A, B, C, G and Figure S9). In contrast, activation of BMPR1A-mediated signaling failed to restore dermal activation of Smad1/5/8 in the *Gpr177^K14^*/Tg-pmes-*caBmpr1a* mutants (Figure 6D, E, F, H and Figure S9), consistent with non-cell autonomous effects of BMP signaling on the spinous layer (Figure 3). These findings strongly suggest that BMP signaling functions primarily in the dermis, through the canonical pathway, to regulate downstream signaling molecules that act back on the epidermis to control epidermal stratification.

### FGF signaling acts downstream of the BMP pathway in epidermal stratification

**Figure 7:**
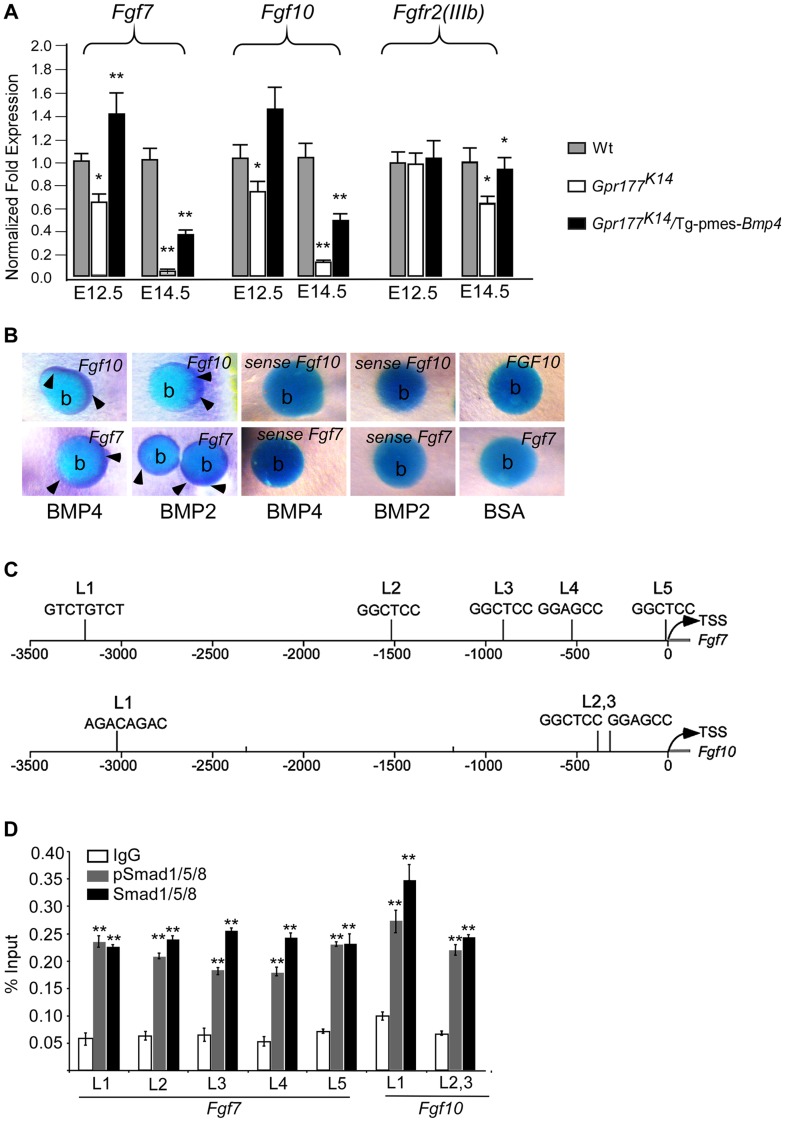
Genetic transduction of BMP signaling via FGF7/FGF10 in dermis to promote epidermal stratification. (A) Quantitative real-time RT-PCR performed on mRNA isolated from dissected autopod skin at E12.5 (n = 5) and E14.5 (n = 3) reveals reduced expression of *Fgf7* and *Fgf10*. Note that significantly reduced expression of *Fgfr2-IIIb* isoform is detected at E14.5. *, *P*<0.05; **, *P*<0.01, compared with wild type controls. Data are represented as mean ± SD and are representatives of at least 3 independent experiments. (B) Implanted BMP2 and BMP4 protein-soaked beads in dermis explants induces the expression of Fgf7 and Fgf10 (arrowheads) around the protein beads, as shown by whole-mount in situ hybridization. Data are representatives of at least three independent experiments. b: protein beads. sense: sense riboprobes. (C) Schematic diagram shows location of Smad1/5/8-binding sites of the Fgf7 and Fgf10 regulatory region. L1-L5 represents Smad1/5/8-binding site with GGMGCC or GTCTGTCT sequence [41], [42]. (D) Quantitative levels of ChIP assays were analyzed by real-time PCR. ChIP assays were performed with Smad1/5/8 or pSmad1/5/8 antibody. Immunoprecipitated DNA was amplified by real-time PCR and presented as a percentage of input. The data shown are representative of two independent experiments with similar results. Error bar represent standard deviations of the PCR reactions performed in triplicate. Student's *t*-test was used for statistical analysis. **, P<0.01. L, locus. TSS, Transcription start site. See also Figure S6.

**Figure 8:**
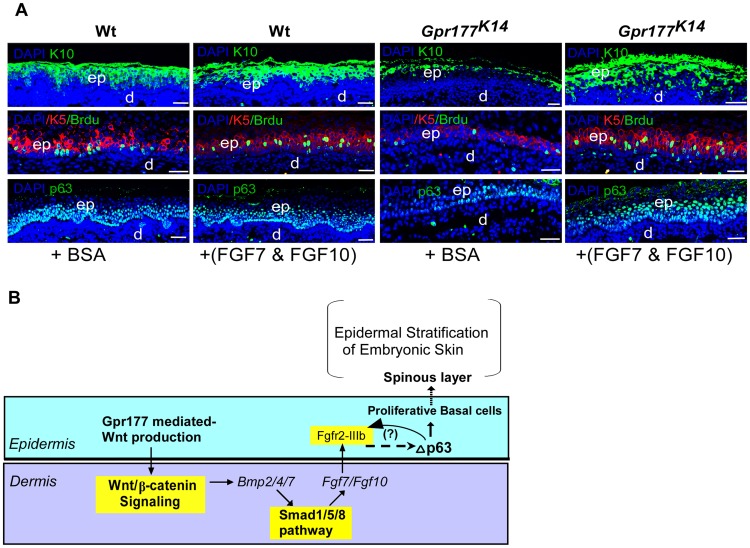
FGF7/FGF10 in dermis promotes the embryonic epidermal stratification in response to Wnt signaling. (A) Epidermal stratification was promoted in FGF7/FGF10 proteins treated and organ-cultured skin of *Gpr177^K14^* mutant. Immunostaining using antibodies against KRT10 (green), KRT5 (red), and p63(green)shows that supplemented FGF7/FGF10 proteins in culture medium have no effect on epidermal stratification of wild type controls. Immunostaining on KRT1 and KRT10 for spinous layer, KRT5 for basal layer, and p63 shows that hypoplastic development of epidermis in the *Gpr177^K14^* mutant skin is significantly attenuated by exogenously supplied FGF7 and FGF10 in organ culture. Note an increase in numbers of both BrdU incorporated KRT5 cells and p63 expressing cells in FGF7/FGF10 treated skin explants from *Gpr177^K14^* mice. ep: epidermis; d: dermis. Bars: 50 µm. (B) Proposed model for a genetic hierarchy of WNT-BMP-FGF7/FGF10 signaling axis in regulating the embryonic epidermal stratification.

We next sought to identify the downstream mediators of BMP signaling on epidermal stratification. FGF signaling came to our attention because several FGF ligands are known to be expressed exclusively in the dermal cells [22], [39], and knockout of *Fgf10* or its receptor FGFR2-IIIb leads to epidermal hypoplastic defects [23], similar to that seen in *Gpr177^K14^* mutants (Figure 1). Using real time RT-PCR analysis, we found that *Gpr177* deficiency significantly diminishes the expression of *Fgf7* (KGF) and *Fgf10* (Figure 7A), both working in concert to activate downstream signaling via FGFR2-IIIb [24], [26], [28], [40]. Furthermore, the reduced expression of *Fgf7* and *Fgf10* in *Gpr177^K14^* mutants was restored by transgenic *Bmp4* expression (Figure 7A and Figure S10A–C). Interestingly, a decrease in the expression of epidermal-specific *Fgfr2IIIb* was not significantly detected in the *Gpr177^K14^* mutant at the early stage, but was observed at E14.5 (Figure 7A), suggesting an indirect consequence of activation. This reduction of *Fgfr2IIIb* expression was restored in *Gpr177^K14^*Tg-pmes-*Bmp4* mice (Figure 7A). In vitro beads implantation assays further demonstrated that exogenously applied BMP2 or BMP4 was able to induce the expression of *Fgf7* (17/20 in BMP2 implants and 15/21 in BMP4 implants) and *Fgf10* (15/19 in BMP2 implants and 22/25 in BMP4 implants) in the dermal explants of *Gpr177^K14^* mice (Figure 7B), supporting our hypothesis that FGF signaling acts downstream of the Wnt/BMP regulatory axis. To further determine if both *Fgf7* and *Fgf10* are transcription targets of pSmad1/5/8 signaling, we tested potential binding of pSmad1/5/8 to the regulatory region of *Fgf7* and *Fgf10* by in vivo chromatin immunoprecipitation (ChIP) assays using embryonic limb skin samples. We utilized five sets of oligos pairs (see Methods and Materials) that amplify five potential binding sites of Smad1/5/8 [41], [42] in the regulatory regions of *Fgf7* (Figure 7C) and two sets of oligo pairs for the binding sites in that of *Fgf10* (Figure 7C). Quantitative PCR showed that after immunoprecipitation of linked chromatin there was specific enrichment of Smad to a DNA fragment that corresponds to one of potential sites with antibodies against either pSmad1/5/8 or Smad1/5/8 compared to IgG controls (Figure 7D). Thus, ChIP results strongly support the notion that in embryonic limb skin of mouse in vivo, activated Smad1/5/8 is present in the regulatory regions of *Fgf7* and *Fgf10* loci. To further demonstrate the involvement of FGF signaling in epidermal stratification, organ culture analysis was performed. The wild type and *Gpr177^K14^* skin explants were supplemented with BSA as controls, or with exogenous FGF7 and FGF10. Immunostaining of keratinocyte markers was carried out 48 hours in organ culture. Although the wild type explants exhibited minimal response to the exogenous FGF7 and FGF10, the mutant explants exhibited increased thickness of the spinous layer, elevated number of KRT5-expressing mitotic cells, as well as enhanced expression of p63 in the presence of FGF7 and FGF10 (Figure 8A and Figure S10). Our results thus uncover a functional requirement of the Wnt/BMP/FGF signaling axis as well as their signaling interplay across the epidermis and dermis to orchestrate epidermis stratification.

## Discussion

The Wnt, BMP, and FGF signaling pathways play critical roles in the embryonic development of the skin [11], [23], [24], [25], [43], [44]. Recent studies using mouse models with *Wls*/*Gpr177* deletion have shown that Wnt secreted from the epidermis is essential for the dermal activation of the canonical Wnt pathway and activation of BMP signaling during hair follicle induction [14], [33]. However, how Wnt, BMP, and FGF pathways interact in the genetic networking that regulates the epidermal stratification during embryogenesis remains unclear. Here we used a transgenic *Bmp4* mouse line to successfully rescue the defective epidermal stratification of *Gpr177^K14^* mice. We dissect the sequential relationship and signaling crosstalk by which these key pathways interact and mediate epidermal stratification. Based on our results, we propose a genetic hierarchy model that integrates Wnt, BMP, and FGF signaling in the regulation of epidermal stratification (Figure 8B). In this model, a BMP/Smad1/5/8/FGF7/10 signaling cascade in the dermis is activated by epidermal Wnts and feedbacks to regulate basal cell proliferation and the subsequent epidermal stratification. Although the specificity of the Cre mouse line used in this study allows us to present this molecular circuit based on data from the limb skin, our observations from the dorsal skin suggest that the molecular responses involved in this model do not bias the body regions (Figure S1, S4, S6, S8A).

Our in vivo results showed that the proliferating basal cells expressing ΔNp63 were targets of the epidermal Wnt signal, and failed expression of ΔNp63 accounts for the hypoproliferation of these basal cells in the absence of epidermal Wnt. It is consistent with the functional importance of p63 in controlling basal cell proliferation of epidermal development and homeostasis [5], [10], [18], [45], suggesting that sustained expression of Wnt pathway regulated ΔNp63 is critical in maintaining the capability of basal keratinocytes to form the stratified epidermis in the developing mouse embryo.

ΔNp63 has been implicated in the developmental program of epidermal stratification through several mechanisms, including aymmetric division of basal cells and cell cycle exit of intermediate suprabasal cells [3], [5], [46], [47]. Although the basal layer lacking epidermal Wnt failed to maintain the proliferative capability of ΔNp63-expressing cells to form a normal spinous layer, the developmental events of epidermal stratification do take place normally, as evidenced by the occurrence of the asymmetric basal cell division to form intermediate mitotic keratinocytes and the replacement of these cells by post-mitotic keratinocytes in spite of a thinned spinous layer. Hence, our studies suggest that the mechanism by which epidermal production of Wnt affects the vertical expansion of the epidermis underlying the ΔNp63-governed basal keratinocytes is independent of both initiation of the intermediate keratinocytes and cell cycle exit for epidermal differentiation.

Notably and interestingly, unlike the effects of autocrine Wnt signaling on the interfollicular epidermal stem cells (IFESCs) of adult skin [48], loss of epidermal Wnt production in the embryonic skin in our study is not associated with premature differentiation of basal cells. Given the evidence of the embryonic epidermis as a tissue source for activation of β-catenin/Wnt signaling in the dermis of the developing skin [14], [33], there appears to exist a functional requirement for paracrine Wnt signaling in the maintenance of proliferative basal cells in epidermal stratification of embryonic skin.

Epidermal deletion of *Gpr177* disrupts the canonical Wnt signaling in the dermis [14], [33] at E13.5, prior to the formation of the intermediate keratinocytic layer and maturation of the spinous layer [3], [5], [9]. Subsequently, expression of *Bmp2*, *Bmp4*, *Bmp7*, *Fgf7*, and *Fgf10*, critical for epidermal development [21], [22], [39], [44], [49], is specifically disrupted in the dermis [14], indicating that Wnt signaling functions upstream of these signals. BMP signaling appears to act downstream of Wnt signaling to mediate Wnt function, because activation of BMP signaling (Smad1/5/8 signaling) in the dermis of *Gpr177^K14^* mutants successfully rescues the development of epidermal stratification and underlying molecular events. Irrespective of the contribution of BMPR1A and BMPR1B [36], [50], canonical BMP signaling is activated both in the epithelium and in the dermal mesenchyme of developing skin [51], [52], [53]. Our findings show that while the expression of transgenic *Bmp4* is activated in the epidermis of *Gpr177^K14^* mice, the activation of canonical BMP signaling in the dermis enable it to rescue epidermal stratification, suggesting that BMP/Smad1/5/8 signaling in the dermis mediates Wnt signaling to control basal cell proliferation, consistent with the recognized role of balanced BMP signaling in the maintenance of epidermal stem cells, progenitor cell differentiation, and hair follicle induction [1], [21], [36], [44], [54]. Based on the specific activation of Smad1/5/8 pathway by non-cell autonomous transgenic BMP4 seen in the dermis of *Gpr177^K14^* mutants, we suggest that the downstream signaling feedback mechanism is required for the regulation of epidermal basal cells.

Given that loss of epidermal Wnt production at least partially phenocopies the epidermal defects in mice lacking Fgfr2-IIIb [23], the expression of *Fgf7* and *Fgf10* in the dermis is directly dependent on the presence of BMP/Smad1/5/8 signaling in the dermis in response to Wnt signaling. This implicates FGF7/10 as the downstream mediator for canonical BMP signaling in the dermis for the maintenance of basal cell proliferation. This hypothesis is supported by our skin organ culture experiments where exogenously applied FGF7/FGF10 are sufficient to functionally attenuate the reduction of proliferative basal cells and to rescue the hypoplastic spinous layer of the *Gpr177^K14^* skin, consistent with the function of FGF7 and FGF10 in epidermal development [25], [28], [55]. It would be interesting to see if other keratinocyte mitogens such as EGF can exert similar rescue functions as the FGFs in future investigations. Nevertheless, we propose that in normal stratification of embryonic epidermis, FGF7 and FGF10 secreted from the dermis diffuse to the epidermis to mediate feedback regulation of Wnt and BMP/Smad1/5/8 signaling, which is required for the maintenance of proliferative keratinocytes in the basal layer through modulation of ΔNp63 [56], [57]. Consistent with previous studies that showed FGFR2 is a transcription target of p63 in the epidermis [56], [58], our quantitative RT-PCR results showing the down-regulation of Fgfr2-IIIb at the late stages of epidermal development further support a role of Fgfr2 signaling acting downstream of p63 in epidermal development. Nonetheless, our data suggest that the FGF7/FGF10 function as feedback factors to epidermis, but cannot rule out the possibility of involvement of additional feedback mechanisms [58], [59] between FGF7/10, Fgfr2, and p63 in the epidermis. However, the mechanism of how FGF7/10 signaling feedbacks to the epidermis and positively regulates ΔNp63 to maintain the proliferative basal cells remains unknown and warrants future studies.

In the adult skin, interfollicular epidermal basal cells, unlike hair follicles, proliferate throughout animal life. Recent studies on subtle genetic deletions by Millar and colleagues [60] have distinguished that Wnt/β-catenin signaling contribute to the mechanism controlling interfollicular epidermal cell (IFE) proliferation in the postnatal skin rather than the long-term maintenance of IFE stem cells. In embryonic skin development, our current study supports the notion that the epidermal Wnt initiates mesenchymal responses in the dermis by activating a BMP-FGF signaling cascade. This activation is crucial for the feedback regulations that control the stratification processes in the interfolliclular epidermis, indicating a profound effect of Wnt on signaling interplays across the epithelium and the mesenchyme in orchestrating the basal cell proliferation during epidermal stratification.

## Materials and Methods

### Generation and analysis of mutant mice

Mice carrying *Gpr177* floxed allele [30] was crossed with *K14-Cre* transgenic mice [61] to generate mice with epidermal loss-of-function of *Gpr177* (*Gpr177^K14^*). A *Dermo1-Cre* mouse was crossed to *Gpr177 floxed* allele to delete *Gpr177* in dermal compartment of the skin [14]. *TOPOGAL* reporter [62], BATGAL reporter [63], *R26R* reporter, *Dermo1-Cre* mice, and transgenic *K14-Cre* mice were purchased from The Jackson Laboratory, Maine. Generation of transgenic Tg-pmes-*Bmp4* and Tg-pmes-*caBmpr1a* mice has been described previously, in which the transgenic allele expresses *Bmp4* (or *caBmpr1a*) and *Gfp* (Green fluorescent protein) simultaneously via an IRES (Internal Ribosome Entry Site) [34], [35]. Animal experimental protocols were approved by The Animal Committee of Hangzhou Normal University, China.

### Histology, in situ hybridization, RNA extraction, and real-time RT-PCR

Embryo collection, histology, and in situ hybridization for whole-mount and on sections were performed as previously described [32].

For real-time RT-PCR, embryonic autopods were dissected and treated with 0.1% collegenase to separate the dermal and epidermal compartments. RNA extraction using RNA isolation kit (ambion, RNAqueous-4RNA) and real-time RT-PCR analysis for RNA expression were performed as previously described [32]. The primers: QAxin2: 5′-ACGCAC- TGACCGACGATT-3′ and 5-AAGGCAGCA- GGTTCCACA-3′; QFzd1: 5′-GAGTTCTGGACCAGTAATCCGC-3′ and 5′- ATGAGCCCGT- AAACCTTGGTG-3′; QLef1: 5′- AACGAGTCCGAAATCATCCCA-3′ and 5′- GCCAGAGTA- ACTGGAGTAGGA-3′; QTcf4: 5′-GATGGGACTCCCTATGACCAC-3′ and 5′- GAAAGGGTT- CCTGGATTGCCC-3′; QBmp2: 5′- TCTTCCGGGAACAGATACAGG-3′ and 5′- TGGTGTCC- AATAGTCTGGTCA-3′; QBmp4: 5′-GACTTCGAGGCGACACTTCTA-3′ and 5′- GAATGA- CGGCGCTCTTGCTA-3′; QBmp7: 5′-AGGGCTTCTCCTACCCCTAC-3′ and 5′- GGTGGTAT- CGAGGGTGGAAGA-3′; Q18S: 5′- GAAACGGCTACCACATCC-3′ and 5′- ACCAGAC- TTGCCCTCCA-3′; QDkk1: 5′- GACCTGCTACGAGACCTGGA-3′ and 5′- CTGGAGAGGG- TATGGTTGCC-3′; QFgf7: 5′-CAGAACAAAAGTCAAGGAGCAACCG-3′ and 5′- GTCGCTCGGGGCTGGAACAG-3′; QFgf10: 5′- TCAGCGGGACCAAGAATGAAG-3′ and 5′-CGGCA- ACAACTCCGATTTCC-3′; QFgfr-IIIb: 5′- CCTCGATGTCGTTGAACGGTC-3′ and 5′- CAGCATCCATCTCCGTCACA-3′. QTg-Bmp4: 5′- GGGCTGGCCATTGAGGTGAC-3′ and 5′-ATGGCGACGGCAGTTCTTATTCTT-3′. QTg-caBmpr1a: 5′- TAATAACACATGCATAACTAAT-3′ and 5′-GCTTTTGGTGAATCCTTGCA -3′.

### BrdU labeling and apoptosis assays

Cell proliferation rate was measured by BrdU incorporation as previously described [32]. Briefly, timed pregnant mice were injected intraperitoneally with BrdU solution at a dosage of 3 mg/100 g of body weight using BrdU Labeling and Detection Kit (Roch Applied Science) 30 minutes prior to embryo collection. Cell apoptosis was detected with TUNEL assay kit (Roche Applied Science). At least 4 embryonic limbs for each genotype were fixed in 4% paraformaldehyde and processed for at 5–7 µm paraffin sections for immunofluorescence analysis according to manufacturer's instructions.

### Immunohistochemistry

Embryonic limb were fixed in 4% PFA for 30 minutes, washed several times in PBS, and then processed for either paraffin sections or cryostat sections. For cryostat sections, samples were treated for in 5% sucrose and 15% sucrose, 2 hours each, in 30% sucrose. For 2–3 days. Samples were embedded in OCT and sectioned at 20 µm. To conduct immunohistochemical staining, sections were washed 3 times in PBST (0.1%Triton X-100/PBS), then blocked in 5% BSA for 30 minutes, and incubated with primary antibodies diluted with 5% BSA at 4°C overnight in a humid chamber. Sections were subsequently washed in PBST, 3 times for 10 minutes each. Secondary antibodies (1∶1000) and DAPI (1∶500) diluted in 5% BSA were applied for 30 minutes in the dark. Following application of secondary antibodies, the sections were washed several times with PBST, for 10 minutes for each, mounted with Mowiol (Sigma) and stored at 4°C. Primary antibodies used in this study were commercially purchased from Abcam, as detailed below: Cytokeratin 5 (ab24647), Cytokeratin 10 (ab9025), Cytokeratin 1 (ab24643), Filaggrin (ab24584), Loricrin (Ab24722), Anti-laminin (ab14055), p63 (ab53039). Antibody against ΔN-p63 was purchased from Santa Cruz (sc-8609), Antibody against BrdU was purchased from Roche (19691800) and antibody against pSmad1/5/8 purchased from Cell Signaling. Antibody against FGF10 was purchased from Santa Cruz (sc-7375).

### Quantification of cell proliferation and antibody-positive cells

For quantification of proliferation, BrdU-positive cells were counted (n = 3–7 limb samples, ≥15 consecutive fields at 40× magnification) and calculated as a percentage of antibody labeled cells and total nuclear stained cells (DAPI positive) otherwise within a defined arbitrary area. For quantification of pSmd1/5/8-positive cells in either the epidermis or the underlying dermis in Figure 6G–H, the numbers of pSmad1/5/8 positive cells in every 300 DAPI positives were counted and calculated as a percentage (n = 3–5 limb samples, ≥15 fields at 40× magnification for each genotype). For quantification of epidermal p63-positive cells in Figure 5, p63-posive cells were counted and calculated in similar way as described above (n = 3 limb samples for each geneotype). Statistical significance was determined using Student's *t*-test.

### Implantation of protein beads and culture of embryonic epidermal explants

Embryonic limbs were dissected from embryos at E13.5 and dorsal skin was separated manually using fined forceps and placed dorsal upward onto a Nucleopore membrane in a culture plate with a central well. Protein beads were soaked with BMP2 (100 ng/µl, R&D), BMP4 (100 ng/µl, R&D), BSA (l00 ng/µl). Explants were cultured at 37°C for 24 hours after implantation of beads onto explants.

Skin organ culture of the dorsal-autopod was conducted using a modification of a previously published procedure [24]. Briefly, dorsal skin portions were dissected from embryonic autopods (hands/feet) at late E13.5 with the assistance of 0.1% collagenase treatment. Skin explants were placed epidermal side up onto a Nucleopore filter (Whitman, pore-size 0.7 µm) that was coated with rat tail collagen type 1 (Sigma) in an organ culture plate with a central well, and cultured in DMEM without serum in 5% CO2 for 72 hours. Protein mixtures of recombinant FGF7 (R&D) and FGF10 (R&D) were applied onto DMEM medium at a final concentration of 250 ng/µl each, and the protein-containing media were replaced every 12 hours. In parallel experiments, BSA was applied onto DEME medium at the same concentration of proteins as control. Organ-cultured skin samples were fixed with 4% PFA and processed for paraffin sections for either immunohistochemistry or H&E staining.

### Whole-mount X-gal staining and electronic microscopy

β-Gal staining for both whole-mount and cryostat sections were performed with commercial purchased Kit (Roche) according to manufacturer's instructions. For electronic microscopic analyses, embryonic limbs were fixed in 2.5% glutaraldehyde and dehydrate through graded ethanol and acetone. Samples were processed according to standard protocols

### Chromatin immunoprecipitation

Limb skin tissues from E13.5 mouse embryos were cut into small pieces, and then rinsed in 1% formaldehyde/PBS for 30 min on ice for cross-linking. The cross-linking reaction was stopped by adding glycine to a final concentration of 0.125 M and rotating for 5 min. The crosslinked tissues were ground by Dounce tissue grinder in tissue lysis buffer from Magna ChIP G Tissue Kit. Lysed cells were collected by spin at 10,000× g for 5 min. The pelleted cells were resuspended in 200 µl of Micrococcal nuclease buffer per 30 mg of the pelleted cells. The resuspended cells were digested with 1 µl of Micrococcal nuclease (New England Biolabs) at 37°C for 20 min. Then the reaction was stopped by adding EDTA to a final concentration of 50 mM and followed by sonication on ice at 30 W for 12 pulses of 1 second on, 3 seconds off to further disrupt and release chromatins. Chromatin immunoprecipitation was performed with antibody against Smad1/5/8 (Santa Cruz, sc-6031), pSmad1/5/8 (Cell signaling technology, 9511) or normal rabbit IgG (Beyotime, A7016) using Magna ChIP G Tissue Kit (Millipore) according to the user manual. For the detection of the immunoprecipitated *Fgf7* and *Fgf10* promoter region, eluted DNA was used as template for quantitative real time PCR analysis with primers specific for Smad-binding sites [41], [42]. Real-time PCR was performed in triplicate using SsoFast EvaGreen Supermix with CFX96 Real-Time PCR Detection System (Bio-Rad Laboratories).

Primers: Fgf7-L1:5′-CTCCATCCTGGTTTTCCTCC-3′ and 5′-GAATAGGACACAGGAAGACAG-3′; Fgf7-L2:5′-AACCTGCTCAGTGACATTCC-3′ and 5′-ACTACAGAATGCCCAGTCTC-3′; Fgf7-L3:5′-TTAGGGTGGTGATACGATGG-3′ and 5′-CTTTCCAGCCTGAGCTTGTG-3′; Fgf7-L4:5′-AGCTGAGCCATGGGGAAGTA-3′ and 5′-GGCTGAGAAGACCTAGTTTC-3′; Fgf7-L5:5′-TTGCTTCCAATGAGGTCAGC-3′ and 5′-GATTTTCTCCGTGTGTGAGC-3′; Fgf10-L1:5′-GGCCATAGAAACAGAGCATG-3′ and 5′-GCTTCAGATTAGAATGGTACC-3′; Fgf10-L2,3:5′-GCAATTAGCAGGAGCTGCAG-3′ and 5′-GATGCCTTTG- CTCTGAGCTG-3′.

## Supporting Information

Figure S1 K14-Cre activity is consistent in epidermis of embryonic limb but inconsistent in body skin. (A–B) Whole-mount in situ hybridization shows RNA expression of Gpr177 in the developing mouse limb. (C–D) *R26R/K14-Cre* embryonic autopods are stained by X-gal at E12.5 (C) and E13.5 (D) and sectioned along dashed lines, showing consistent Cre activity in the limb epidermis (inserts). (E) A section image shows inconsistent Cre activity along dorsal body of *R26R/K14-Cre* embryo at E12.5. (F–G, F′–G′) Immunofluorescence (red) of Gpr177 expression in the epidermis (arrows) and the underling dermis (arrowheads) in the dorsal body skin between E12.5 and E13.5. Note an incomplete deletion of Gpr177 in the skin at both stages (F′ and G′). (H, H′) H&E staining shows inconsistent defect in epidermal thickness of the dorsal body skin of *Gpr177^K14^* mice at E16.5. Dashed lines demarcate the boundary between the epidermis and the dermis. Bars: 50 µm. (I–J, I′–J′) Immunohistochemistry shows expression of KRT1 (red) for the spinous layer, and KRT5 (red) for the basal layer of the body skin at E18.5. Bars: 50 µm.
(TIF)

Figure S2 Histology of *Gpr177^K14^* mutant epidermis. (A–D) H&E staining shows hypoplastic limb skin of *Gpr177^K14^* mice (B), rescued thickness of epidermis in *Gpr177^K14^*/Tg-pmes-*Bmp*4 mice (C), and failed rescued epidermis of in *Gpr177^K14^*/Tg-pmes-*caBmpr1*a mice (D). Bars: 50 µm (E) Quantification of epidermal thickness (µm) in wild type controls and *Gpr177^K14^* autopod skin at E18.5. (**, *P*<0.01, n = 5). Data are represented as mean ± SD. (F–H) Transmission electronic microscope images of epidermis. Note that the reduced thickness of spinous layer (s) in *Gpr177^K14^* mice is rescued in *Gpr177^K14^*/Tg-pmes-*Bmp*4 mice. d: dermis; b: basal layer; s: spinous layer; g: granular layer. Bars: 5 µm.
(TIF)

Figure S3 Cell death assays. (A–D) TUNEL assay performed on the sections of autopod skin at E16.5 shows that cell apoptosis (green) is comparable among distinct genotypes. DAPI is stained as blue.
(TIF)

Figure S4 Deletion of Gpr177 in embryonic epidermis leads to ablation of Wnt/β-catenin signaling in dermis of dorsal skin. (A–D) X-Gal staining on sections of dorsal limbs and dorsal body show BATGAL activity in the dermal mesenchyme at E12.5 and E13.5. (E–F and E′–F′) TOPGAL activity in the dermal mesenchyme of the dorsal body is still detectable at E13.5 but lack at E14.5.
(TIF)

Figure S5 Deletion of Gpr177 in dermis did not alter the thickness of epidermis. (A–B) Histological images of dorsal skin in wild type control (*Gpr177^fx^/Dermo1-Cre*) at E14.5 and E15.5. (C–D) Sections of dorsal skin in dermis-specific Gpr177 deletion (*Gpr177^fx/fx^/Dermo1-Cre*) mice at E14.5 and E15.5.
(TIF)

Figure S6 A graph showing Kyoto Encyclopedia of Genes and Genomes (KEGG) biological pathways for downregulated genes in the *Gpr177^K14^* limb sample. The bar plot shows the top ten Enrichment score (−log10 (P value)) values of the significant enrichment pathways. Note that individual genes may be present in more than one category.
(TIF)

Figure S7 Expression of *Bmps* in body skin development requires epidermal Gpr177. (A–F, A′–F′) In situ hybridization reveals the reduced transcripts of *Bmp2*, *Bmp4*, and Bmp7 in epidermis (arrows) and dermis (arrowheads) of *Gpr177^K14^* embryonic body skin at E14.5 and E16.5, as compared to wild type controls.
(TIF)

Figure S8 p63 expression in basal cells during epidermal stratification in *Gpr177^K14^* mice. (A) Expression of *p63* in the body skin of (arrows) *Gpr177^K14^* mice is reduced, as compared to wild type controls. (B–J) Pan-p63 expression in limb skin is reduced in basal cells of *Gpr177^K14^* mice between E13.5 and E15.5 (white arrowheads in B,E,H) compared to wild type controls (red arrowheads in A,D,G), and the defective p63 expression is rescued in epidermis of *Gpr177^K14^*/Tg-pmes-*Bmp4* mice (red arrowheads in C,F,I). Note that p63 is also expressed in intermediated cells and appears comparable in mice of all three genotypes (white arrows in A–F). It is highlighted in epidermis dual-stained by anti-p63 and anti-KRT10 (G–I). (K–P) Immunostaining shows that lack of TA-p63 in wild type epidermis during epidermal stratification.
(TIF)

Figure S9 Transgenic pmes-*Bmp4* reactivates Smad1/5/8 signaling in the dermal mesenchyme in *Gpr177^K14^*. (A–B) Immunofluorescence detections for anti-phosphorylated-Smad1/5/8 (p-Smad1/5/8, green) on sections of autopods at E16.5. P-Smad1/5/8 activity (white arrowheads) is preferentially decreased in the dermis of limb skin in *Gpr177^K14^* mice and increased in dermis of *Gpr177^K14^*/Tg-pmes-*Bmp4* mice (A). Dash lines demarcate the border of epidermis and dermal mesenchyme. Immunofluorescence staining using antibodies against p-Smad1/5/8 on sections of dorsal autopod skin shows that p-Smad1/5/8 activity is only increased in epidermis of *Gpr177^K14^*/Tg-pmes-*caBmpr-1a* mice (B). epi: epidermis; dm: dermis. (C–D) Quantification of pSmad1/5/8 positive cells in the epidermis and dermis of *Gpr177^K14^*/Tg-pmes-*Bmp4* (C) and *Gpr177^K14^*/Tg-pmes-*caBmpr-1a* mice (D) at E16.5. Data are represented as mean ± SD. *, P<0.05; **, *P*<0.01, n = 2.
(TIF)

Figure S10 The expression of Fgf10 in the dermis is activated by Smad1/5/8/BMP signaling and is sufficient for epidermal stratification. (A–C) Immunostaining shows expression of Fgf10 is reduced in the *Gpr177^K14^* dermis and restored in the *Gpr177^K14^*/Tg-pmes-*Bmp4* dermis. (D–E) Supplement of FGF7/FGF10 protein (E) but not BSA protein (D) in skin organ culture increases epidermal thickness of *Gpr177^K14^* mice. H&E staining on sections of skin. Bars: 50 µm. (F) Quantification of percentage of BrdU incorporated KRT-5 cells in the epidermis. Supplement of FGF7/FGF10 protein but not BSA protein in limb skin organ culture increase basal cell proliferation in *Gpr177^K14^*. The experiment was conducted at least 3 times with a minimum of 6 skin samples in each group. Data are represented as mean ± SD. **, *P*<0.01. (G) Statistical analysis shows the ratios of p63-positive cells in the epidermis. Data are represented as mean ± SD. **, *P*<0.01.
(TIF)

Table S1 The top ten enriched Kyoto Encyclopedia of Genes and Genomes (KEGG) biological pathways for genes down-regulated in *Gpr177^K14^* sample.
(DOCX)

Table S2 Differentially expressed pathway genes included in Supplementary Table S1. The 73 genes are arranged alphabetically by gene name. LC represents wild type sample; LM represents *Gpr177^K14^* sample.
(DOCX)
