# Supplementary figures and images for: BMP-FGF Signaling Axis Mediates Wnt-Induced Epidermal Stratification in Developing Mammalian Skin
Source: PLoS Genet. 2014 Oct 16;10(10):e1004687. doi: 10.1371/journal.pgen.1004687 (PMC4199507; doi:10.1371/journal.pgen.1004687)

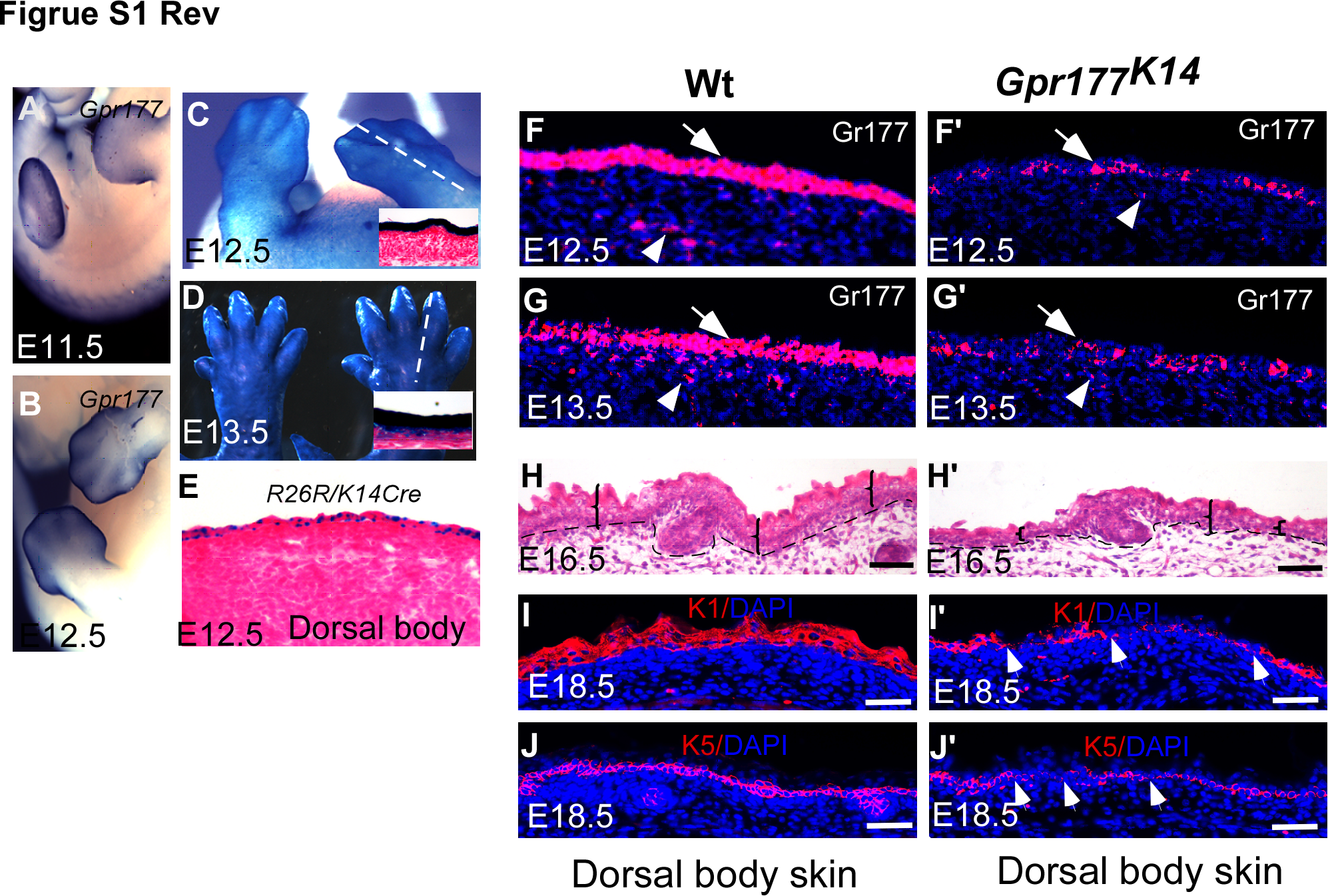

Supplement: Figure S1 — K14-Cre activity is consistent in epidermis of embryonic limb but inconsistent in body skin. (A–B) Whole-mount in situ hybridization shows RNA expression of Gpr177 in the developing mouse limb. (C–D) R26R/K14-Cre embryonic autopods are stained by X-gal at E12.5 (C) and E13.5 (D) and sectioned along dashed lines, showing consistent Cre activity in the limb epidermis (inserts). (E) A section image shows inconsistent Cre activity along dorsal body of R26R/K14-Cre embryo at E12.5. (F–G, F′–G′) Immunofluorescence (red) of Gpr177 expression in the epidermis (arrows) and the underling dermis (arrowheads) in the dorsal body skin between E12.5 and E13.5. Note an incomplete deletion of Gpr177 in the skin at both stages (F′ and G′). (H, H′) H&E staining shows inconsistent defect in epidermal thickness of the dorsal body skin of Gpr177K14 mice at E16.5. Dashed lines demarcate the boundary between the epidermis and the dermis. Bars: 50 µm. (I–J, I′–J′) Immunohistochemistry shows expression of KRT1 (red) for the spinous layer, and KRT5 (red) for the basal layer of the body skin at E18.5. Bars: 50 µm. (TIF) [file pgen.1004687.s001.tif]

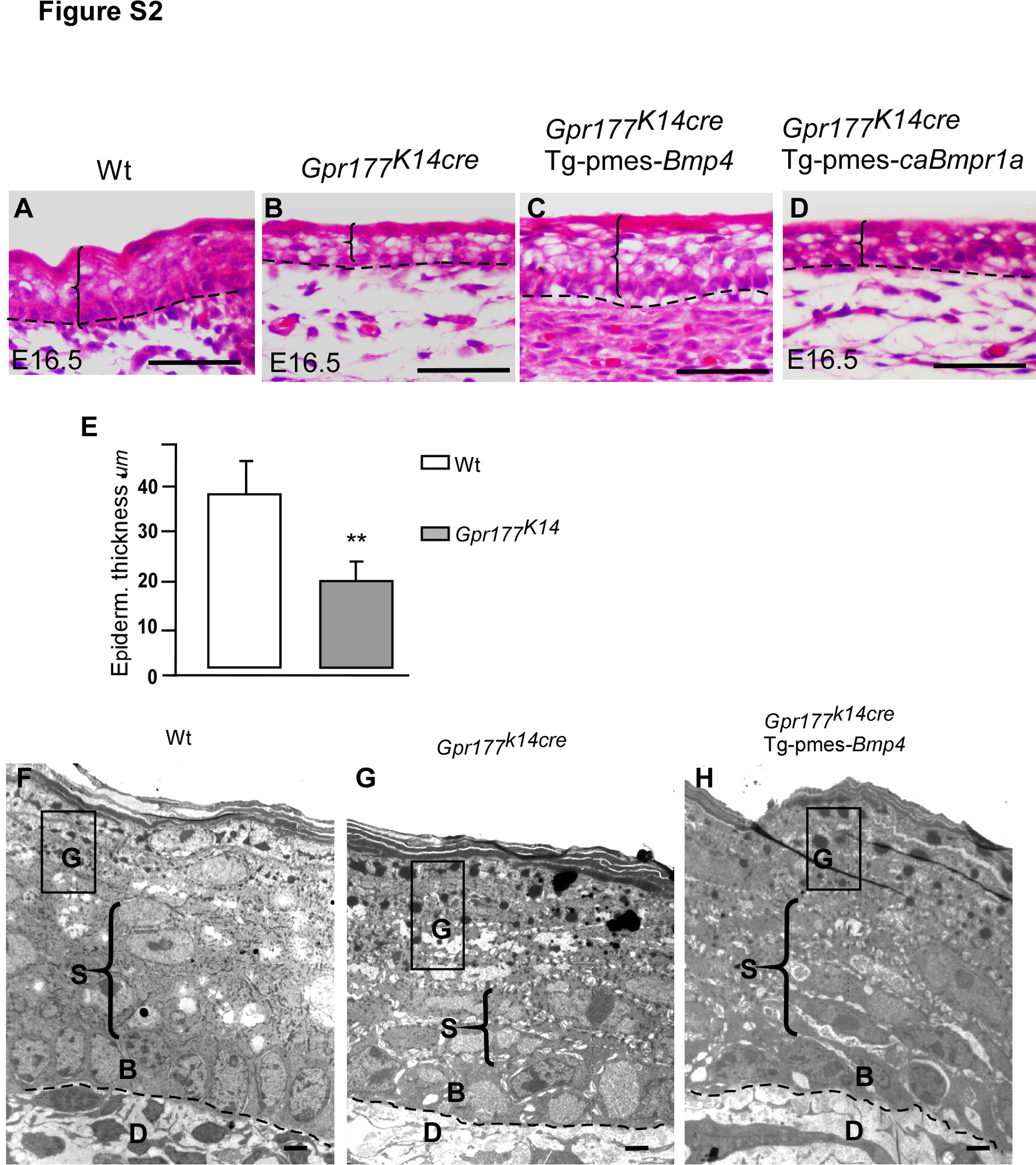

Supplement: Figure S2 — Histology of Gpr177K14 mutant epidermis. (A–D) H&E staining shows hypoplastic limb skin of Gpr177K14 mice (B), rescued thickness of epidermis in Gpr177K14/Tg-pmes-Bmp4 mice (C), and failed rescued epidermis of in Gpr177K14/Tg-pmes-caBmpr1a mice (D). Bars: 50 µm (E) Quantification of epidermal thickness (µm) in wild type controls and Gpr177K14 autopod skin at E18.5. (**, P<0.01, n = 5). Data are represented as mean ± SD. (F–H) Transmission electronic microscope images of epidermis. Note that the reduced thickness of spinous layer (s) in Gpr177K14 mice is rescued in Gpr177K14/Tg-pmes-Bmp4 mice. d: dermis; b: basal layer; s: spinous layer; g: granular layer. Bars: 5 µm. (TIF) [file pgen.1004687.s002.tif]

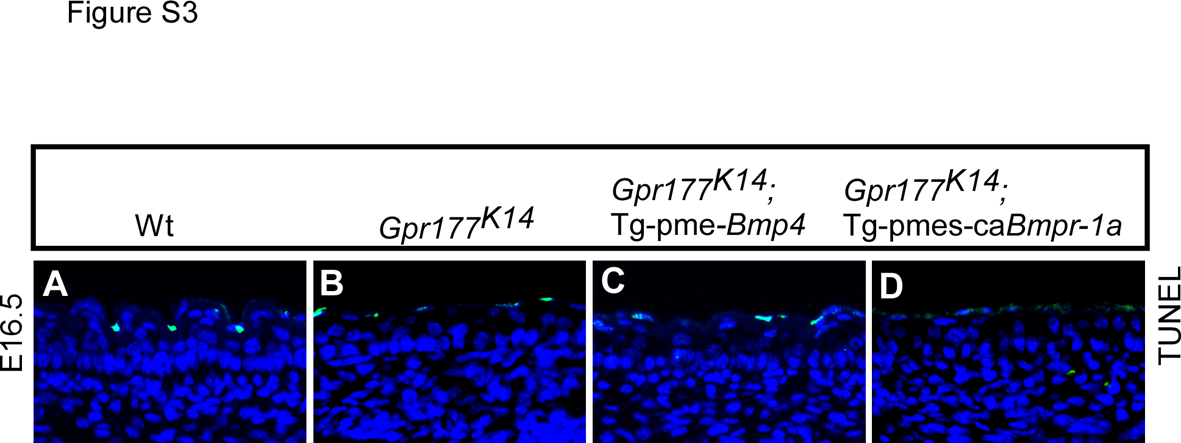

Supplement: Figure S3 — Cell death assays. (A–D) TUNEL assay performed on the sections of autopod skin at E16.5 shows that cell apoptosis (green) is comparable among distinct genotypes. DAPI is stained as blue. (TIF) [file pgen.1004687.s003.tif]

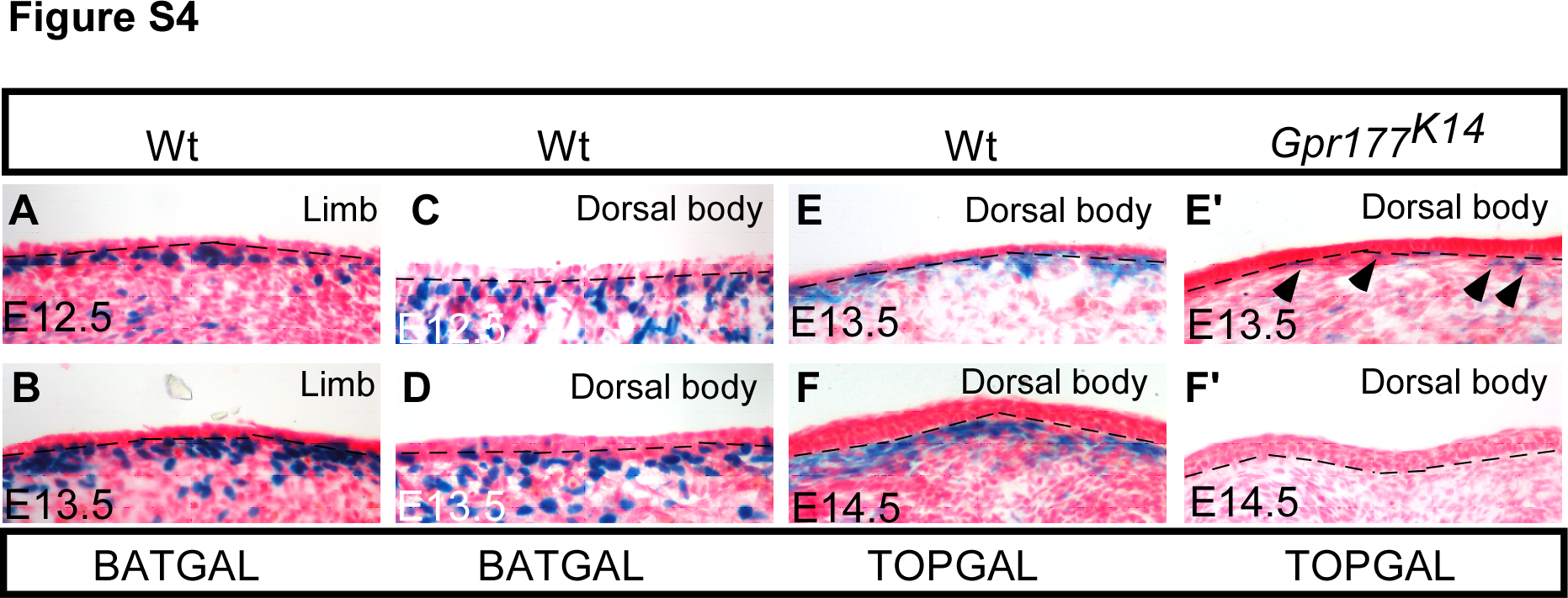

Supplement: Figure S4 — Deletion of Gpr177 in embryonic epidermis leads to ablation of Wnt/β-catenin signaling in dermis of dorsal skin. (A–D) X-Gal staining on sections of dorsal limbs and dorsal body show BATGAL activity in the dermal mesenchyme at E12.5 and E13.5. (E–F and E′–F′) TOPGAL activity in the dermal mesenchyme of the dorsal body is still detectable at E13.5 but lack at E14.5. (TIF) [file pgen.1004687.s004.tif]

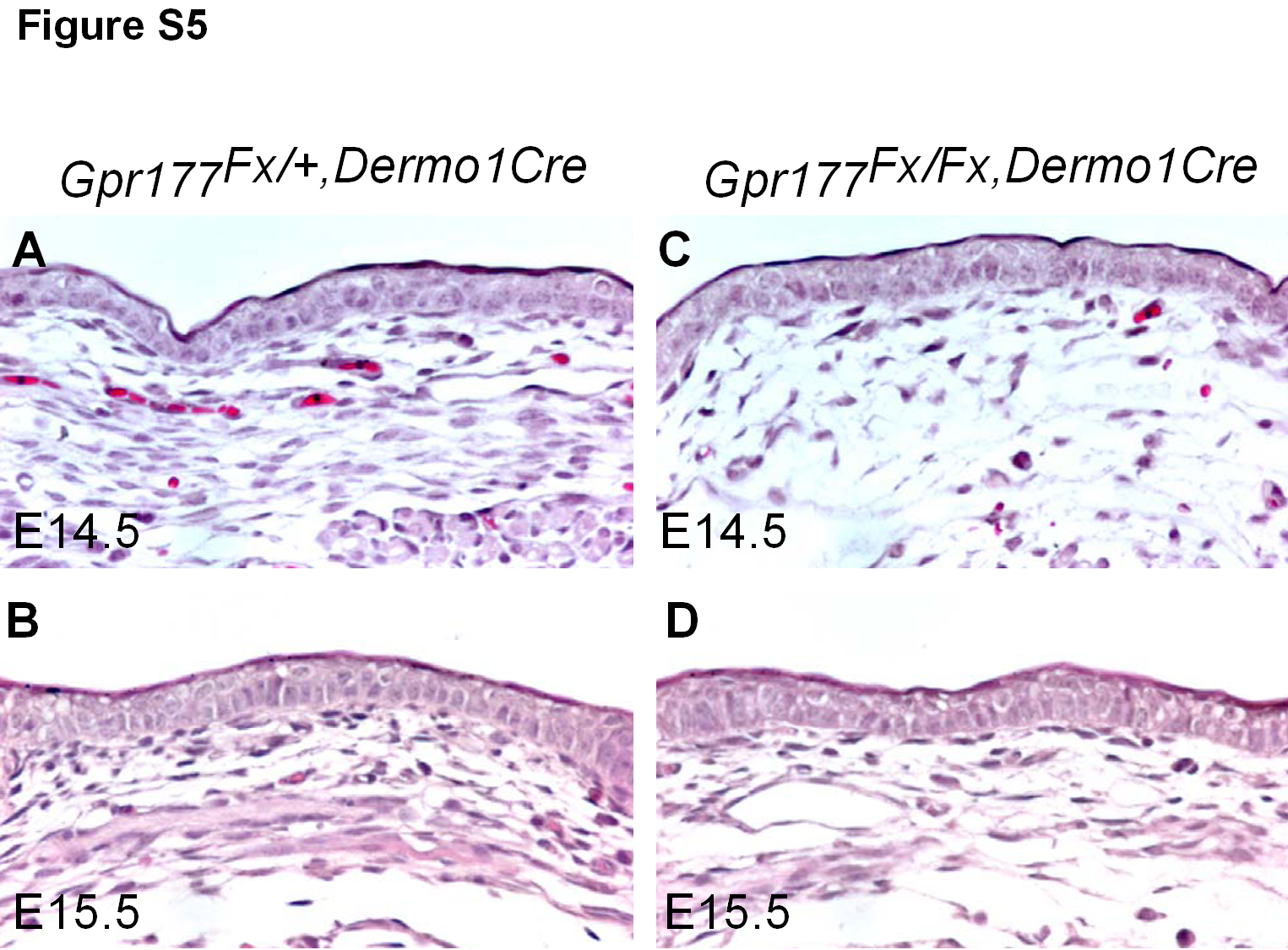

Supplement: Figure S5 — Deletion of Gpr177 in dermis did not alter the thickness of epidermis. (A–B) Histological images of dorsal skin in wild type control (Gpr177fx/Dermo1-Cre) at E14.5 and E15.5. (C–D) Sections of dorsal skin in dermis-specific Gpr177 deletion (Gpr177fx/fx/Dermo1-Cre) mice at E14.5 and E15.5. (TIF) [file pgen.1004687.s005.tif]

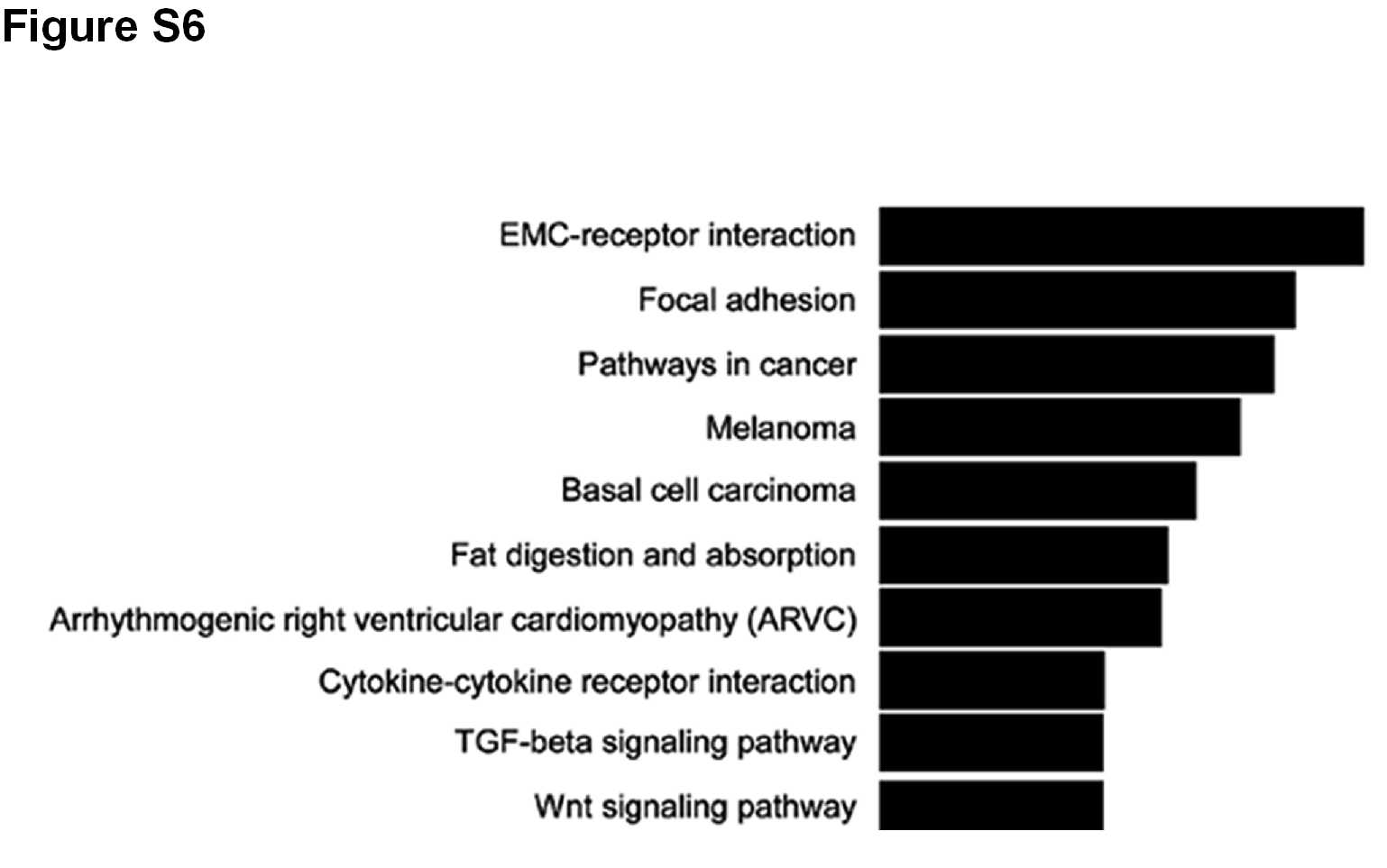

Supplement: Figure S6 — A graph showing Kyoto Encyclopedia of Genes and Genomes (KEGG) biological pathways for downregulated genes in the Gpr177K14 limb sample. The bar plot shows the top ten Enrichment score (−log10 (P value)) values of the significant enrichment pathways. Note that individual genes may be present in more than one category. (TIF) [file pgen.1004687.s006.tif]

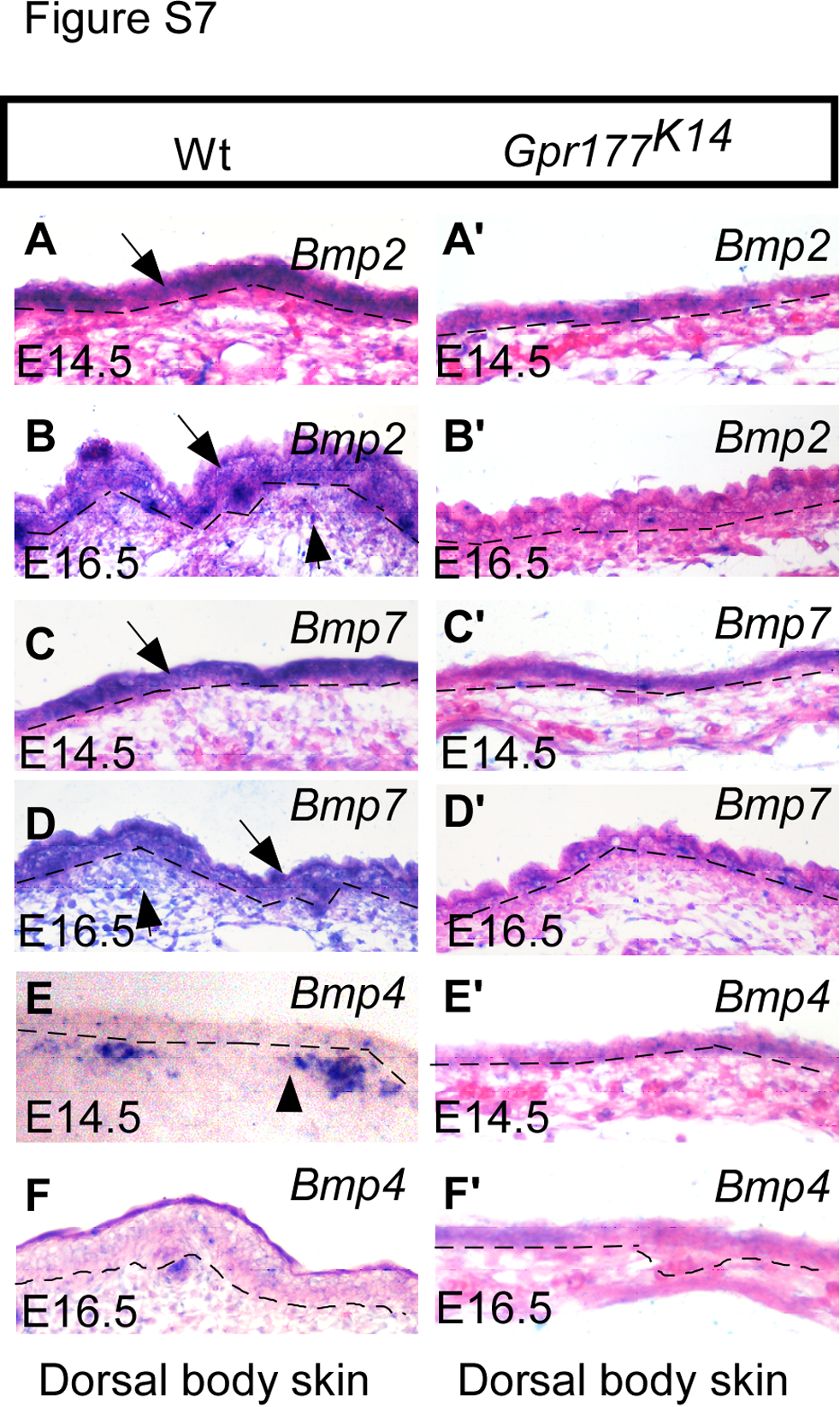

Supplement: Figure S7 — Expression of Bmps in body skin development requires epidermal Gpr177. (A–F, A′–F′) In situ hybridization reveals the reduced transcripts of Bmp2, Bmp4, and Bmp7 in epidermis (arrows) and dermis (arrowheads) of Gpr177K14 embryonic body skin at E14.5 and E16.5, as compared to wild type controls. (TIF) [file pgen.1004687.s007.tif]

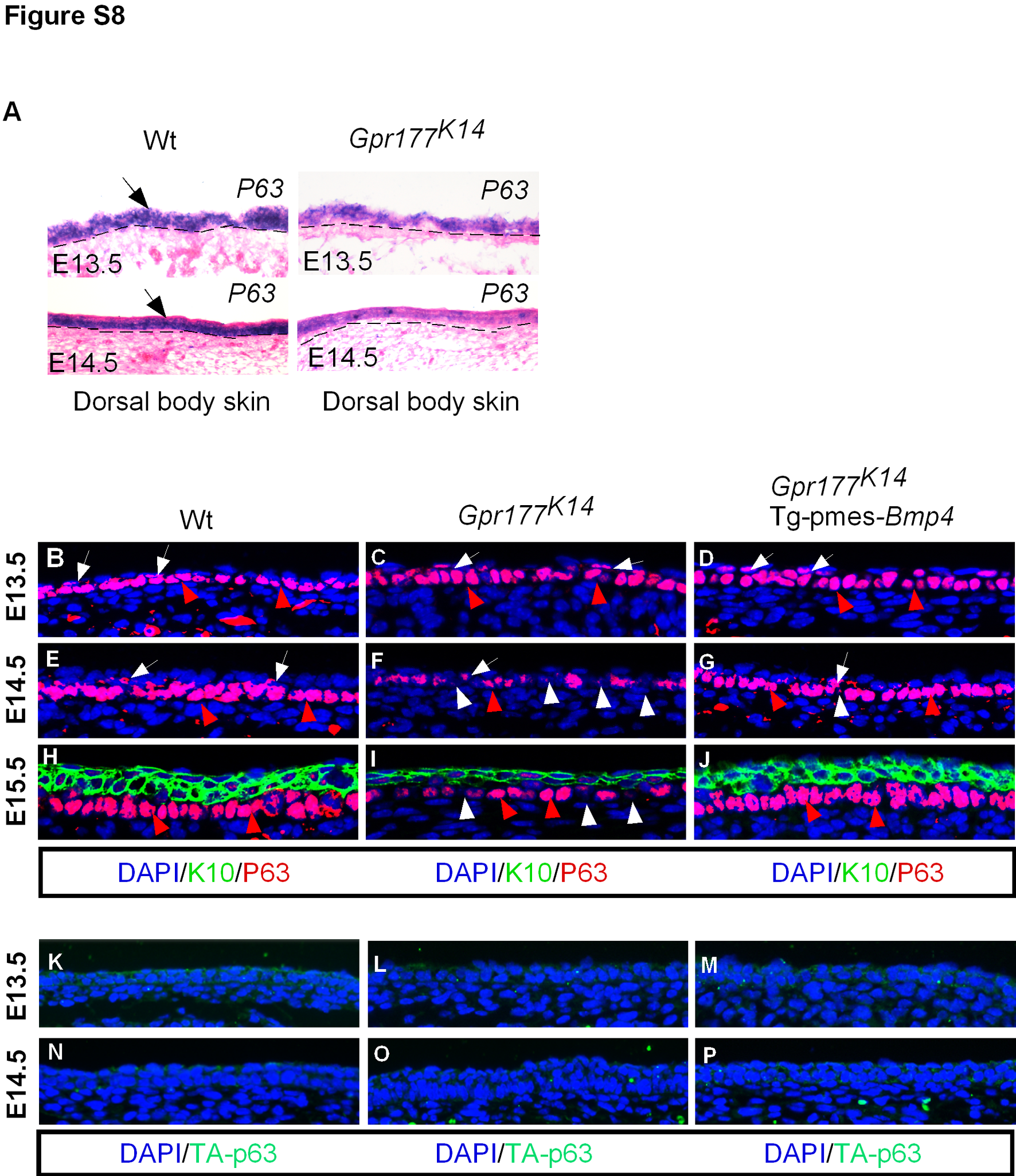

Supplement: Figure S8 — p63 expression in basal cells during epidermal stratification in Gpr177K14 mice. (A) Expression of p63 in the body skin of (arrows) Gpr177K14 mice is reduced, as compared to wild type controls. (B–J) Pan-p63 expression in limb skin is reduced in basal cells of Gpr177K14 mice between E13.5 and E15.5 (white arrowheads in B,E,H) compared to wild type controls (red arrowheads in A,D,G), and the defective p63 expression is rescued in epidermis of Gpr177K14/Tg-pmes-Bmp4 mice (red arrowheads in C,F,I). Note that p63 is also expressed in intermediated cells and appears comparable in mice of all three genotypes (white arrows in A–F). It is highlighted in epidermis dual-stained by anti-p63 and anti-KRT10 (G–I). (K–P) Immunostaining shows that lack of TA-p63 in wild type epidermis during epidermal stratification. (TIF) [file pgen.1004687.s008.tif]

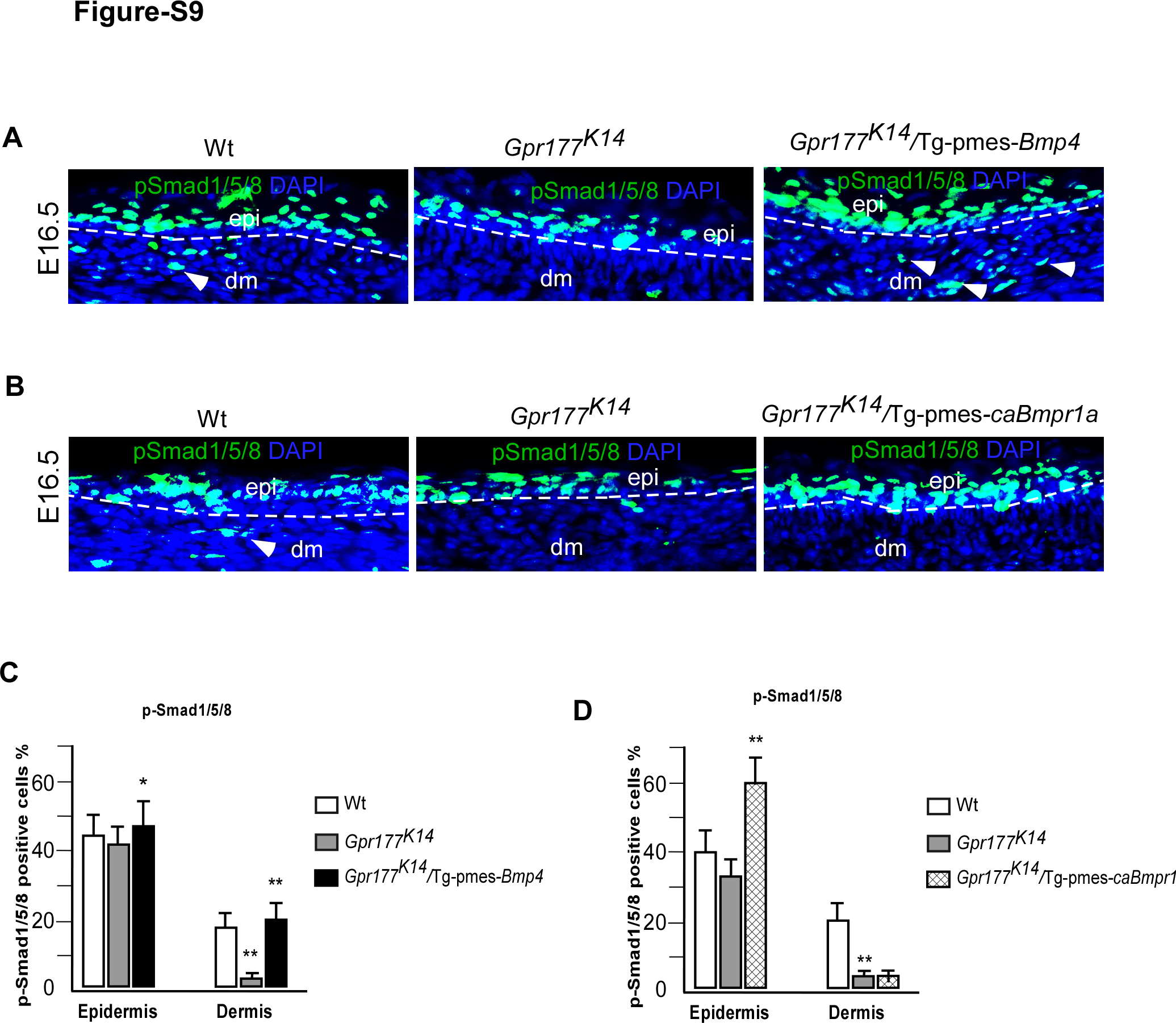

Supplement: Figure S9 — Transgenic pmes-Bmp4 reactivates Smad1/5/8 signaling in the dermal mesenchyme in Gpr177K14. (A–B) Immunofluorescence detections for anti-phosphorylated-Smad1/5/8 (p-Smad1/5/8, green) on sections of autopods at E16.5. P-Smad1/5/8 activity (white arrowheads) is preferentially decreased in the dermis of limb skin in Gpr177K14 mice and increased in dermis of Gpr177K14/Tg-pmes-Bmp4 mice (A). Dash lines demarcate the border of epidermis and dermal mesenchyme. Immunofluorescence staining using antibodies against p-Smad1/5/8 on sections of dorsal autopod skin shows that p-Smad1/5/8 activity is only increased in epidermis of Gpr177K14/Tg-pmes-caBmpr-1a mice (B). epi: epidermis; dm: dermis. (C–D) Quantification of pSmad1/5/8 positive cells in the epidermis and dermis of Gpr177K14/Tg-pmes-Bmp4 (C) and Gpr177K14/Tg-pmes-caBmpr-1a mice (D) at E16.5. Data are represented as mean ± SD. *, P<0.05; **, P<0.01, n = 2. (TIF) [file pgen.1004687.s009.tif]

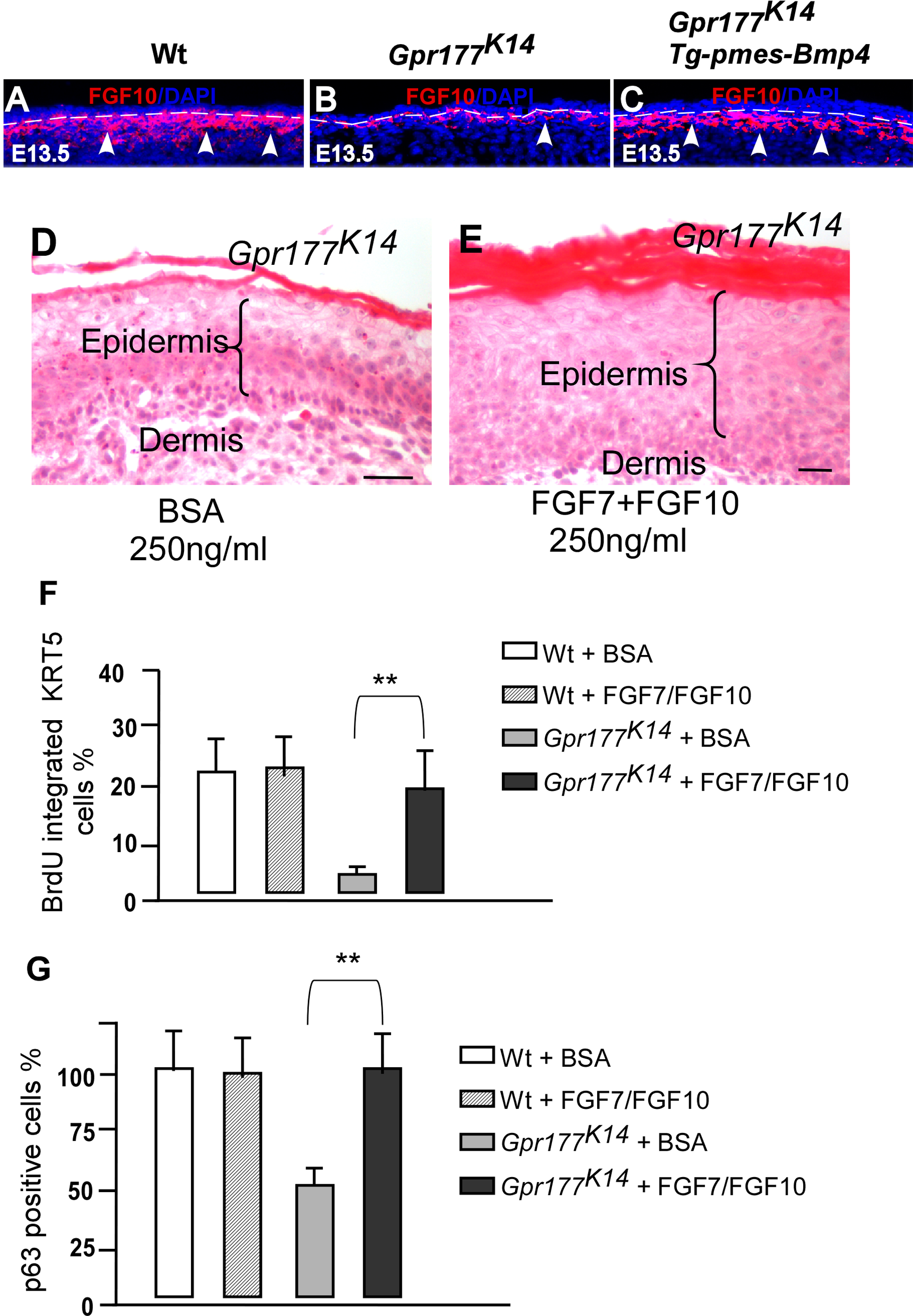

Supplement: Figure S10 — The expression of Fgf10 in the dermis is activated by Smad1/5/8/BMP signaling and is sufficient for epidermal stratification. (A–C) Immunostaining shows expression of Fgf10 is reduced in the Gpr177K14 dermis and restored in the Gpr177K14/Tg-pmes-Bmp4 dermis. (D–E) Supplement of FGF7/FGF10 protein (E) but not BSA protein (D) in skin organ culture increases epidermal thickness of Gpr177K14 mice. H&E staining on sections of skin. Bars: 50 µm. (F) Quantification of percentage of BrdU incorporated KRT-5 cells in the epidermis. Supplement of FGF7/FGF10 protein but not BSA protein in limb skin organ culture increase basal cell proliferation in Gpr177K14. The experiment was conducted at least 3 times with a minimum of 6 skin samples in each group. Data are represented as mean ± SD. **, P<0.01. (G) Statistical analysis shows the ratios of p63-positive cells in the epidermis. Data are represented as mean ± SD. **, P<0.01. (TIF) [file pgen.1004687.s010.tif]
