# Supplementary material for: BMP-FGF Signaling Axis Mediates Wnt-Induced Epidermal Stratification in Developing Mammalian Skin
Source: PLoS Genet. 2014 Oct 16;10(10):e1004687. doi: 10.1371/journal.pgen.1004687 (PMC4199507; doi:10.1371/journal.pgen.1004687)
Supplement: Table S1 — The top ten enriched Kyoto Encyclopedia of Genes and Genomes (KEGG) biological pathways for genes down-regulated in Gpr177K14 sample. (DOCX) [file pgen.1004687.s011.docx]

**Table S1.**

| **Pathway**  **ID** | **KEGG**  **Pathway** | **Fisher P-value** | **Selection**  **Counts** | **Selection**  **Size** | **Count** | **Size** | **FDR** | **Enrichment**  **Score** | **Genes** |
| --- | --- | --- | --- | --- | --- | --- | --- | --- | --- |
| mmu04512 | ECM-  receptor interaction | 0.000608536 | 11 | 278 | 86 | 6913 | 0.06815431 | 3.215713 | COL1A1/COL1A2/COL4A6/COL6A2/COL6A3/ITGA2B/ITGA5/ITGB4/RELN/SPP1/THBS2 |
| mmu04510 | Focal adhesion | 0.001090355 | 18 | 278 | 200 | 6913 | 0.06815431 | 2.962432 | COL1A1/COL1A2/COL4A6/COL6A2/COL6A3/FLT1/FYN/ITGA2B/ITGA5/ITGB4/MYL7/MYLK/PDGFD/PDGFRB/PIK3CB/RELN/SPP1/THBS2 |
| mmu05200 | Pathways in cancer | 0.001345151 | 25 | 278 | 326 | 6913 | 0.06815431 | 2.871229 | AXIN2/BAX/BMP2/BMP4/CCDC6/COL4A6/CUL2/CYCS/E2F2/FGF7/FGF9/FGFR1/FZD1/HHIP/ITGA2B/KIT/MITF/PAX8/PDGFRB/PIK3CB/RB1/RUNX1/RUNX1T1/TCF7/WNT5B |
| mmu05218 | Melanoma | 0.002223185 | 9 | 278 | 72 | 6913 | 0.08448104 | 2.653024 | E2F2/FGF7/FGF9/FGFR1/MITF/PDGFD/PDGFRB/PIK3CB/RB1 |
| mmu05217 | Basal cell carcinoma | 0.00611762 | 7 | 278 | 55 | 6913 | 0.1859757 | 2.213417 | AXIN2/BMP2/BMP4**/**FZD1/HHIP/TCF7/WNT5B |
| mmu04975 | Fat digestion and absorption | 0.008718624 | 6 | 278 | 45 | 6913 | 0.2025715 | 2.059552 | ABCG5/AGPAT2/PLA2G12A/PLA2G4B/PNLIPRP2/SCARB1 |
| mmu05412 | Arrhythmogenic right ventricular cardiomyopathy | 0.009328949 | 8 | 278 | 74 | 6913 | 0.2025715 | 2.030167 | CACNB2/CACNG1/DMD/GJA1/ITGA2B/ITGA5/ITGB4/TCF7 |
| mmu04060 | Cytokine-cytokine receptor interaction | 0.02024678 | 17 | 278 | 246 | 6913 | 0.2838806 | 1.693644 | BMP2/CCL21A/CCL27A/CXCR4/FLT1/IL17RB/IL9/INHBA/KIT/LEPR/NGFR/PDGFD/PDGFRB/RELT/TNFRSF13C/TNFRSF18/TNFRSF19 |
| mmu04350 | TGF-beta signaling pathway | 0.02043581 | 8 | 278 | 85 | 6913 | 0.2838806 | 1.689608 | BMP2/BMP4/DCN/FST/INHBA/PPP2R1B/SMAD5/THBS2 |
| mmu04310 | Wnt signaling pathway | 0.02094991 | 12 | 278 | 154 | 6913 | 0.2838806 | 1.678818 | AXIN2/CAMK2G/CSNK1A1/DKK2/FZD1/NKD1/NKD2/PPP2R1B/PPP2R5A/TCF7/WIF1/WNT5B |
| Pathway ID stands for Pathway identifiers used in KEGG  Fisher P-value stands for the enrichment p-value of the Pathway ID used Fisher's exact test  Selection Counts stands for the Count of the differentially expressed genes’ entities directly associated with the listed PathwayID  Selection Size stands for the total number of the differentially expressed genes’ entities  Count stands for the count of the chosen background population genes’ entities associated with the listed Pathway ID  Size stands for the total number of chosen background population genes’ entities  FDR stands for the false discover rate of the Pathway ID  Enrichment Score stands for the Enrichment Score value of the Pathway ID, it equals -log10(P-value) | | | | | | | | | |
