# Supplementary material for: BMP-FGF Signaling Axis Mediates Wnt-Induced Epidermal Stratification in Developing Mammalian Skin
Source: PLoS Genet. 2014 Oct 16;10(10):e1004687. doi: 10.1371/journal.pgen.1004687 (PMC4199507; doi:10.1371/journal.pgen.1004687)
Supplement: Table S2 — Differentially expressed pathway genes included in Supplementary Table S1. The 73 genes are arranged alphabetically by gene name. LC represents wild type sample; LM represents Gpr177K14 sample. (DOCX) [file pgen.1004687.s012.docx]

**Table S2.**

| **Fold change**  **(LM vs LC)** | **LC**  **(normalized intensity)** | **LM**  **(normalized intensity)** | **ACCESSION** | **GENE**  **NAME** | **SYNONYM** |
| --- | --- | --- | --- | --- | --- |
| -3.057 | 176.7277 | 57.82001 | BC106766 | Abcg5 | AW112016,MGC123400,sterolin-1 |
| -2.354 | 145.16167 | 61.661297 | AK090271 | Agpat2 | 2510002J07Rik,AV000834,BSCL,BSCL1,LPAAB,LPAAT-beta |
| -4.794 | 327.35117 | 68.28442 | AK084644 | Axin2 | Axi1,Axil,Conductin |
| -2.250 | 564.14276 | 250.73065 | AY095934 | Bax | - |
| -2.096 | 1234.6855 | 589.18567 | BC100344 | Bmp2 | AI467020,Bmp2a |
| -2.279 | 535.46045 | 234.97078 | BC013459 | Bmp4 | Bmp-4,Bmp2b,Bmp2b-1,Bmp2b1 |
| -3.159 | 225.26723 | 71.312805 | AK082315 | Cacnb2 | AW060387,Cavbeta2,Cchb2,MGC129334,MGC129335 |
| -2.279 | 233.092 | 102.299805 | BC099482 | Cacng1 | MGC117703 |
| -2.043 | 304.59866 | 149.06616 | BC025597 | Camk2g | 5930429P18Rik,Camkg |
| -2.603 | 1143.0398 | 439.1068 | NM_001111121 | Ccdc6 | 2810012H18Rik,AA536681,AA960498,AW061011 |
| -3.809 | 457.90558 | 120.21892 | BC038120 | Ccl21a | 6CKBAC2,6Ckine,ALP,AW987545,CKb9,MGC107632,SCYA21a,SLC,Scya21,Scya21b,Tca4,plt |
| -2.238 | 280.99734 | 125.56259 | BC107226 | Ccl27a | ALP,AW558992,CTACK,CTAK,Ccl27,Ccl27b,ESkine,ILC,MGC130150,PESKY,Scya27,Scya27a,Scya27b |
| -2.380 | 12763.937 | 5363.723 | BC050014 | Col1a1 | Col1a-1,Cola-1,Cola1,Mov-13,Mov13 |
| -2.572 | 22543.016 | 8765.073 | BC007158 | Col1a2 | AA960264,AI325291,Col1a-2,Cola-2,Cola2,oim |
| -2.307 | 320.56592 | 138.95667 | BC057648 | Col4a6 | BB116301 |
| -2.191 | 141.18922 | 64.45009 | AK132540 | Col6a2 | Col6a-2,MGC36205 |
| -3.566 | 4955.689 | 1389.8503 | XM_897036 | Col6a3 | AI507288,Col6a-3 |
| -2.113 | 2575.599 | 1218.7902 | AK019420 | Csnk1a1 | 2610208K14Rik,4632404G05Rik,5430427P18Rik,Csnk1a,MGC29354,MGC30571 |
| -2.168 | 1679.2408 | 774.62537 | AK004875 | Cul2 | 1300003D18Rik,4932411N15Rik,AI327301,KIAA4106,mKIAA4106 |
| -2.315 | 218.7768 | 94.50441 | BC031665 | Cxcr4 | CD184,Cmkar4,LESTR,PB-CKR,PBSF/SDF-1,Sdf1r |
| -2.325 | 509.12552 | 218.99883 | BC034363 | Cycs | - |
| -2.450 | 11966.525 | 4883.405 | BC132521 | Dcn | DC,DSPG2,PG40,PGII,PGS2,SLRR1B,mDcn |
| -3.054 | 304.16345 | 99.595894 | BC096448 | Dkk2 | - |
| -2.361 | 140.43391 | 59.481796 | AK036936 | Dmd | DXSmh7,DXSmh9,Dp427,mdx,pke |
| -2.338 | 163.13992 | 69.767105 | BC062101 | E2f2 | 9230110J10,E130207A07 |
| -2.006 | 158.36156 | 78.9403 | BC052847 | Fgf7 | Kgf |
| -3.322 | 220.5991 | 66.39988 | AK158584 | Fgf9 | Eks |
| -2.022 | 1457.0839 | 720.59216 | AF176552 | Fgfr1 | AW208770,FLG,Fgfr-1,Flt-2 |
| -2.377 | 119.10353 | 50.105606 | BC029674 | Flt1 | AI323757,Flt-1,VEGFR-1,VEGFR1,sFlt1 |
| -2.119 | 351.40857 | 165.8475 | X83377 | Fst | AL033346 |
| -2.052 | 4070.0193 | 1983.4055 | BC032149 | Fyn | AI448320,AW552119,MGC115870 |
| -2.285 | 2095.1584 | 916.80023 | BC053010 | Fzd1 | AW227548,FZ-1 |
| -2.194 | 951.8475 | 433.85333 | AY427559 | Gja1 | AU042049,AW546267,Cnx43,Cx43,Cx43alpha1,Gja-1,Npm1,connexin43 |
| -2.758 | 234.36339 | 84.96711 | AK083190 | Hhip | Hhip1 |
| -2.539 | 129.8652 | 51.150307 | BC026546 | Il17rb | Evi27,IL-17ER,IL-17Rh1,IL17RH1,Il17br |
| -3.120 | 202.6732 | 64.95291 | BC140269 | Il9 | Il-9,P40 |
| -2.050 | 287.6031 | 140.29895 | BC053527 | Inhba | - |
| -3.448 | 193.84557 | 56.221786 | AK161727 | Itga2b | AI172977,CD41,CD41B,GpIIb,alphaIIb |
| -2.155 | 168.29771 | 78.091194 | BC050943 | Itga5 | Cd49e,Fnra |
| -2.334 | 121.28148 | 51.958088 | AK142648 | Itgb4 | AA407042,C230078O20,CD104 |
| -2.419 | 137.48064 | 56.83 | BC052457 | Kit | Bs,CD117,Fdc,Gsfsco1,Gsfsco5,Gsfsow3,SCO1,SCO5,SOW3,Ssm,Tr-kit,W,c-KIT |
| -4.089 | 452.1276 | 110.573494 | BC082551 | Lepr | LEPROT,Leprb,MGC105189,Modb1,OB-RGRP,Obr,db,diabetes,obese-like,obl |
| -2.422 | 190.05208 | 78.4637 | BC108977 | Mitf | MGC124309,MGC124310,bHLHe32,bw,mi,vit,vitiligo,wh |
| -2.578 | 367.82428 | 142.65904 | BC061100 | Myl7 | MLC-2alpha,MLC2a,MYL2A,Mylc2a,RLC-A |
| -2.894 | 451.6255 | 156.08145 | AY237727 | Mylk | 9530072E15Rik,A930019C19Rik,AW489456,KRP,MLCK108,MLCK210,Mlck |
| -2.528 | 187.9665 | 74.34818 | BC038365 | Ngfr | LNGFR,Tnfrsf16,p75,p75NGFR,p75NTR |
| -4.502 | 406.12582 | 90.20061 | BC034838 | Nkd1 | 2810434J10Rik,9030215G15Rik,Nkd |
| -2.822 | 333.1059 | 118.04008 | BC019952 | Nkd2 | 2210403L10Rik,AW212591 |
| -2.025 | 152.15146 | 75.1399 | BC020526 | Pax8 | Pax-8 |
| -2.326 | 127.78041 | 54.946507 | AK141551 | Pdgfd | 1110003I09Rik |
| -3.724 | 413.57498 | 111.06922 | AK143247 | Pdgfrb | AI528809,CD140b,Pdgfr |
| -2.380 | 660.1773 | 277.39722 | BC127073 | Pik3cb | 1110001J02Rik,AI447572,MGC150132,p110beta |
| -2.681 | 259.23163 | 96.682884 | BC026812 | Pla2g12a | 2310004B05Rik,GXII,MGC58884,Pla2g12,Rossy,mGXII-1,mGXII-1-PLA2 |
| -2.199 | 448.4056 | 203.91093 | AK137721 | Pla2g4b | A030011C02Rik,Gm1013,MGC28892 |
| -2.802 | 145.94968 | 52.08431 | BC094923 | Pnliprp2 | PLRP2 |
| -2.538 | 2610.532 | 1028.7554 | BC056218 | Ppp2r1b | 2410091N08Rik,AI790395 |
| -2.016 | 790.7585 | 392.3212 | BC059026 | Ppp2r5a | MGC38798,PR61alpha |
| -3.743 | 261.16162 | 69.77651 | BC096525 | Rb1 | Rb,Rb-1,pRb |
| -2.461 | 126.79276 | 51.527107 | AK042773 | Reln | reeler,rl |
| -2.253 | 142.2299 | 63.119 | BC100310 | Relt | E430021K24Rik,Tnfrsf19l |
| -2.563 | 233.50214 | 91.115585 | NM_001111021 | Runx1 | AI462102,AML1,Cbfa2,Pebp2a2,Pebpa2b |
| -2.426 | 160.33615 | 66.09572 | NM_001111026 | Runx1t1 | Cbfa2t1h,ETO,MTG8 |
| -2.175 | 439.96014 | 202.29106 | BC004656 | Scarb1 | AI120173,CD36,Cd36l1,Cla-1,Cla1,D5Ertd460e,Hlb398,SR-B1,SR-BI,SRBI,Srb1,mSR-BI |
| -2.458 | 138.43512 | 56.316097 | AK139765 | Smad5 | 1110051M15Rik,AI451355,Dwf-C,Madh5,MusMLP |
| -16.182 | 2208.8904 | 136.50267 | AK151069 | Spp1 | AA960535,AI790405,Apl-1,BNSP,BSPI,Bsp,ETA-1,Eta,OP,Opn,Opnl,Ric,Spp-1 |
| -10.712 | 811.3501 | 75.744896 | AK038140 | Tcf7 | AI465550,TCF-1,Tcf1 |
| -2.734 | 628.7779 | 229.96623 | BC053702 | Thbs2 | TSP2,Thbs-2 |
| -7.589 | 396.35086 | 52.2239 | BC104126 | Tnfrsf13c | 2010006P15Rik,BAFF-R,Baffr,Bcmd,Bcmd-1,Bcmd1,Lvis22,MGC123890,MGC123891 |
| -2.283 | 224.94202 | 98.54021 | BC140224 | Tnfrsf18 | AITR,Gitr |
| -2.951 | 356.07797 | 120.65978 | AB040433 | Tnfrsf19 | AL023044,AW123854,TAJ,TAJ-ALPHA,TRADE,Troy |
| -3.257 | 1845.5297 | 566.62134 | BC013268 | Wif1 | AW107799 |
| -2.479 | 126.79276 | 51.150307 | BC010775 | Wnt5b | AW545702,Wnt-5b |
